# Mechanically Robust Mesoporous UiO‐66‐NH_2_/Nanofibrous Aerogel for Organophosphonates Detoxification

**DOI:** 10.1002/advs.202416540

**Published:** 2025-03-16

**Authors:** Mai O. Abdelmigeed, Muhammed Ziauddin Ahmad Ebrahim, Vahid Rahmanian, John J. Mahle, Gregory W. Peterson, Saad A. Khan, Gregory N. Parsons

**Affiliations:** ^1^ Chemical and Biomolecular Engineering North Carolina State University 911 Partners Way Raleigh NC 27695 USA; ^2^ U.S. Army Combat Capabilities Development Command Chemical Biological Center 8198 Blackhawk Road Aberdeen Proving Ground MD 21010 USA

**Keywords:** chemical warfare agents degradation, mesoporous MOFs, nanofiber aerogels, organophosphates hydrolysis, sustainable synthesis process

## Abstract

There is a critical need for novel composite materials for high‐performance chemical filtration and detoxification of organophosphonates (OPs) and other harmful compounds found in nerve agents, pesticides, and industrial processes. In this work, rapid hydrolysis of OPs using high‐surface‐area zirconium‐based MOF‐aerogel composites is demonstrated. Using a unique surfactant‐templated solvothermal synthesis method, mesoporous UiO‐66‐NH_2_ grown on the fibers within a polyacrylonitrile (PAN)/polyvinylpyrrolidone (PVP) nanofibrous sponge can produce a 3D MOF–polymer matrix with a specific surface area of up to 900 m^2^ g^−1^_comp_—almost 2X larger than the highest previously reported values while maintaining robust mechanical integrity. The mesoporous MOF promotes efficient diffusion, and the aerogel matrix provides a high‐surface‐area platform for spill containment. Unlike activated carbon, which adsorb OPs without degradation, the UiO‐66‐NH_2_‐sponges hydrolyze OPs upon water contact, significantly reducing their toxicity. The MOF‐aerogel sponges withstand stresses up to 40 kPa under 70% strain are shown while maintaining exceptional catalytic efficiency, achieving a methyl paraoxon degradation half‐life of 3 min, compared to 15 min for similar microporous MOFs. This innovation accentuates the potential of mesoporous Zr‐MOF aerogels for advanced protection, filtration, and catalysis.

## Graphical abstract

A high‐surface‐area (900 m^2^ g^−1^), ultra‐resilient (40 kPa) metal‐organic framework (MOF)‐polyacrylonitrile (PAN)/polyvinylpyrrolidone (PVP) nanofibrous aerogel (NFA) sponge with mesoporous UiO‐66‐NH₂ enables ultrafast organophosphonate (OP) absorption and detoxification, cutting degradation time to just 3 min—offering a game‐changing solution for chemical filtration and protection.

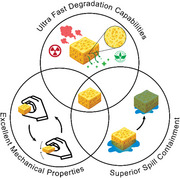

## 1 Introduction

Metal‐organic frameworks (MOFs) are a novel category of porous crystalline materials composed of metal clusters or secondary building units (SBUs) bonded to organic linkers through robust connections.^[^ ^1^ ^]^ These SBUs, comprising inorganic polynuclear clusters, impart MOF structures with stability, directionality, and rigidity.^[^ ^1^ ^]^ The diverse chemical properties of SBUs make MOFs highly adaptable for various applications, including the adsorption of gases and vapors, separation processes, and catalytic reactions facilitated by SBUs.^[^ ^2^, ^3^, ^4^, ^5^, ^6^ ^]^ Organic linkers, typically mono‐, di‐, tri‐, or tetravalent, influence pore size and volume expansion in MOFs. Functionalization of these organic linkers further enhances MOFs' utility in gas separation, energy storage, catalysis, and thermal energy conversion.^[^ ^7^, ^8^, ^9^ ^]^

MOFs have the potential to significantly enhance catalytic activity for the hydrolysis of harmful organophosphonates (OPs) vapor and liquid compounds, including highly toxic chemical warfare agents (CWAs).^[^ ^10^ ^]^ Among these CWAs, GD (soman) and VX (venomous agent X) are nerve agents that act by inhibiting acetylcholinesterase, leading to severe neurological dysfunction and death. Meanwhile, HD (Sulfur Mustard), a vesicant or blister agent, causes severe chemical burns, blisters, and respiratory damage upon dermal or inhalation exposure. These CWAs pose toxicity risks through various routes of exposure, such as oral, dermal, and percutaneous contact.^[^ ^11^ ^]^ Remarkably, even minute quantities, such as a single drop of VX (6 to 10 mg) on the skin,^[^ ^12^ ^]^ or 200 to 1700 mg of tuban (GA), sarin (GB), or GD for a 70 kg individual, can be lethal.^[^ ^13^ ^]^ Additionally, OPs are widely used, constituting approximately 34% of current chemical pesticides.^[^ ^14^ ^]^ Protection against CWAs and related compounds currently relies on their physisorption in highly porous carbon materials.^[^ ^15^ ^]^ However, these carbon materials are predominantly microporous, hindering the diffusion of bulky species. Moreover, a drawback of porous carbon is the potential desorption of toxic compounds under specific conditions due to carbon's negligible reactivity, posing additional exposure risks.^[^ ^16^ ^]^

UiO‐type (University of Oslo) Zr‐based MOFs are considered potential replacements for activated carbon due to their dual capability of adsorption and chemical reactivity resulting primarily from hydrolysis. This hydrolysis process is driven by Zr(IV) metal node centers, which act as strong Lewis acid sites.^[^ ^17^ ^]^ The hydrolysis mechanism involves two steps: first, the nucleophilic addition of OH groups to the organophosphate coordinated to a Zr metal center, forming a phosphorus intermediate; and second, an elimination step.^[^ ^18^ ^]^ The ─NH₂ groups in UiO‐66‐NH₂ contribute to organophosphate hydrolysis through multiple pathways. While the computational studies suggest that the ─NH₂ groups do not act solely as direct nucleophiles, they enhance the local solvent environment, facilitating water activation via Zr‐bound hydroxo groups.^[^ ^19^ ^]^ However, experimental studies propose that ─NH₂ groups can interact directly with the phosphorus atom in OPs, forming transient intermediates that promote P─O bond cleavage. Additionally, their presence improves degradation kinetics through hydrogen bonding, increased substrate affinity, and electronic modulation, highlighting their multifunctional role in the reaction.^[^ ^20^ ^]^

To enhance the catalytic activity of MOFs, strategies such as inducing missing‐linker defects or designing highly porous metal‐organic frameworks (HP‐MOFs)^[^ ^21^ ^]^ with adjustable pore sizes and volumes have been explored. One method involves intentionally introducing missing‐linker defects using a monocarboxylic acid modulator during synthesis. This modulator coordinates with the metal ion to form metal‐oxo clusters, while the alkyl chain introduces structural defects and additional pore space. However, despite these modifications, the resulting MOFs still typically fall within the microporous range (<2 nm).^[^ ^22^ ^]^ Attempts to directly incorporate mesoporous MOFs onto fibers have faced challenges. For example, when mesoporous MIL‐101 (Cr) was integrated onto electrospun polyacrylonitrile (PAN) nanofibers, the outcome was somewhat limited. Despite MIL‐101 (Cr) having a specific surface area surpassing 1900 m^2^ g^−1^_powder_, the resulting composite exhibited a specific surface area of up to 300 m^2^ g^−1^_comp_, representing approximately 15% MOF loading. Moreover, the composite predominantly displayed microporosity.^[^ ^23^ ^]^ This suggests that the MOF‐fabric synthesis process may lead to MOF structure degradation, pore blockage, or both.

In general, the practical use of MOFs is often limited by challenges associated with their use in powder form, which complicates handling and reduces efficiency. Additionally, MOF particles tend to agglomerate, blocking active pores and diminishing performance. To overcome these limitations, researchers have developed mechanically robust macroscopic MOF‐polymer composites.^[^ ^24^, ^25^, ^26^ ^]^ However, traditional 2D composites, such as membranes and thin films, present new challenges, including low throughput, frequent fouling, and limited surface area, making them less suitable for large‐scale applications.^[^ ^9^ ^]^ Aerogels have emerged as a promising alternative for incorporating MOFs, especially for demanding applications like the adsorption of CWAs.^[^ ^27^ ^]^ Their hierarchical porous structure enhances diffusion and mass transfer, while their low density and ultra‐high porosity make them ideal candidates for various advanced applications. Furthermore, aerogels provide the flexibility to fine‐tune porosity and customize functionality through the integration of different MOFs. Recent studies highlight that incorporating a fibrous network significantly improves the mechanical strength of aerogels.^[^ ^28^, ^29^, ^30^ ^]^ Nanofibrous aerogels (NFAs) represent an exciting new class of these materials, consisting of interconnected nanofibers that impart exceptional mechanical robustness. These aerogels are fabricated through the freeze‐drying of homogeneous dispersions of electrospun nanofibers, leading to fiber entanglements that create a bimodal pore distribution, further enhancing their performance.^[^ ^31^, ^32^ ^]^ Previous research on MOF‐based aerogels has largely concentrated on traditional aerogels, which typically involve extracting the solvent phase from a gel‐like network through multiple solvent exchanges, followed by either freeze‐drying or supercritical drying methods.^[^ ^33^, ^34^, ^35^ ^]^ In addition to the cumbersome fabrication steps, such aerogels lack an interconnected network structure, making them brittle and deficient in hierarchical porosity.^[^ ^36^, ^37^ ^]^ In contrast, hybrid aerogels formed by integrating MOFs into nanofibrous matrices combine the best properties of both components offering high surface area, large pore volume, low density, and superior mechanical strength. However, most techniques used to integrate Zr‐based MOFs into aerogels, such as direct mixing,^[^ ^38^, ^39^, ^40^ ^]^ in situ growth,^[^ ^41^, ^42^, ^43^ ^]^ hydrothermal,^[^ ^44^, ^45^ ^]^ and sol‐gel methods,^[^ ^46^ ^]^ yield microporous structures with limited application in catalysis and degradation. Among these, in‐situ growth is mainly employed for adsorption applications, achieving specific surface areas between 10 and 500 m^2^ g^−1^. Despite these advancements, these approaches remain predominantly focused on microporosity and have yet to be effectively tailored for catalytic reactions or the degradation of bulky hazardous compounds.

Our prior research demonstrated the successful integration of mesoporous UiO‐66‐NH_2_ onto fabrics, underscoring its ability to enhance mass transfer and degrade OPs.^[^ ^47^ ^]^ The synthesis process utilized cocamidopropylbetaine (CAPB) as a template, which not only promoted mesochannel formation but also eliminated the need for toxic solvents and harsh acidic conditions, thereby preserving the integrity of the substrate.^[^ ^48^, ^49^ ^]^ The notable increases in surface area and pore volume, along with a significant improvement in degradation efficiency for methyl paraoxon methyl (DMNP), provide a strong basis for further investigation of this material on 3D PAN/polyvinylpyrrolidone (PVPand CDA/silica aerogels. A key aspect of synthesizing mesoporous UiO‐66‐NH_2_ involves the use of an amphoteric surfactant, like CAPB, to facilitate mesopore formation. The interaction between MOF precursors and surfactants is crucial for achieving successful growth of mesoporous MOFs.^[^ ^50^ ^]^ Amphoteric surfactants, characterized by their carboxylate functionality and quaternary ammonium hydrophilic head group, play an essential role in this process. The carboxylate functionality enables strong bonding with metal clusters in the MOFs, while the quaternary ammonium group enhances solubility.^[^ ^51^ ^]^ This dual functionality allows the surfactant to serve both as a template and a coordinating agent, preventing the formation of mesopore‐free MOFs. Additionally, performing the reaction in a water‐based system fosters the creation of rod‐shaped surfactant micelles, guiding MOF growth.^[^ ^51^ ^]^ This aqueous‐phase synthesis overcomes the challenges associated with micelle formation in organic solvents, typically used for MOF synthesis, resulting in well‐defined mesopores.^[^ ^52^ ^]^ This methodology can be adapted for mesoporous MOF‐NFA composites with high OP activity, presenting a promising approach for practical applications.

## 2 Results and Discussions

### 2.1 Powder

In this study, we aim to explore how introducing mesoporosity and a 3D hierarchically porous substrate into Zr‐based MOF‐NFA composites can enhance the hydrolysis rate of CWAs and their simulants. We developed a straightforward and scalable synthesis to create a cost‐effective Zr‐MOF matrix for this application. The mesoporous UiO‐66‐NH_2_ was synthesized through the solvothermal method. The MOF powder, PAN/PVP NFAs, and composite materials were characterized using Fourier transform infrared spectroscopy (FTIR), X‐ray diffraction (XRD), Brunauer–Emmett–Teller (BET) analysis, scanning electron microscopy (SEM), transmission electron microscopy (TEM), thremogravimetric analysis (TGA), and tested for hydrolysis rates of the simulant DMNP, as well as the degradation rates of CWAs like GD and HD.

**Figure 1:**
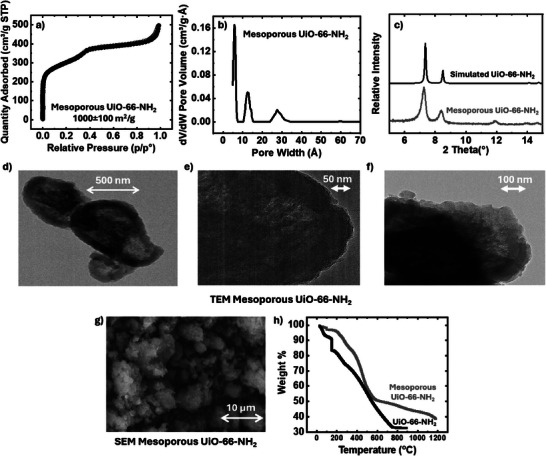
a) N_2_ isotherm of mesoporous UiO‐66‐NH_2_; b) Pore size distribution of mesoporous UiO‐66‐NH_2_; c) XRD of mesoporous UiO‐66‐NH_2_ (gray) simulated UiO‐66‐NH_2_ (black); d–f)TEM images of mesoporous UiO‐66‐NH_2_; g) SEM image of mesoporous UiO‐66‐NH_2;_ and h) TGA of microporous versus mesoporous UiO‐66‐NH_2_.

Starting with the MOF powder characterization, **Figure** **1** presents the results from the N_2_ isotherm, pore size distribution, XRD, SEM, TEM, and TGA analyses of mesoporous UiO‐66‐NH_2_. Figure 1a displays the N_2_‐isotherm of mesoporous UiO‐66‐NH_2_, showing characteristics of both Type I and Type IV isotherms, indicating the presence of both micropores and mesopores. Figure 1b highlights the primary and most pronounced peaks of mesoporous UiO‐66‐NH_2_, observed at 6 and 12 (micropores) and 27 Å (mesopores), with smaller additional peaks extending up to 200 Å. These minor peaks are not shown here due to their lower significance compared to the dominant ones; however, further details are provided in **Table** **1**. In contrast, conventional microporous UiO‐66‐NH_2_ exhibits predominant pore sizes at 6 and 11 Å. The pore sizes in mesoporous UiO‐66‐NH_2_ that are larger than 200 Å, possibly exceeding the detection limit of the non‐local density‐functional theory model (NLDFT) in our BET instrument. The XRD pattern in Figure 1c aligns well with simulated spectra, with diffraction peaks at 2θ values of 7.4°, 8.5°, and 12.0°, corresponding to the (111), (002), and (022) planes of UiO‐66‐NH_2_ crystals.^[^ ^53^ ^]^ Figure 1d–f displays TEM images of UiO‐66‐NH₂ crystals ranging from 0.5 to 1 µm in size, highlighting the mesoporosity of this MOF variant. In Figure 1d, a shell‐like structure is observed, suggesting that defective regions around the particle may have partially collapsed post‐activation, resulting in a denser outer layer in some cases. Figure 1e presents a TEM image at higher magnification, clearly revealing mesopores (≈ 3 nm based on the pore size distribution analysis) at the particle edges and confirming that thermal treatment did not cause their collapse. Additionally, Figure 1f, further illustrates mesopores at the edges and demonstrates that contrast variations between the edge and center depend on multiple synthesis factors. This contrast arises from density differences between the solid MOF framework and the empty pore spaces, aiding in distinguishing mesoporous structures from microporous or non‐porous ones. Figure 1g shows an SEM image of mesoporous UiO‐66‐NH_2_, indicating that the mesoporous MOF crystals tend to cluster, forming a granular structure. Figure 1h presents the TGA profile of mesoporous and microporous UiO‐66‐NH₂, measured under an air atmosphere from ambient temperature to 900 °C. The analysis highlights differences in their thermal stability and organic composition. At 900 °C, the residual mass of mesoporous UiO‐66‐NH₂ is 45%, whereas the microporous counterpart retains only 32.5%, indicating a higher inorganic fraction in the mesoporous sample. This 12.5% difference suggests a lower organic linker content in the mesoporous structure, likely due to structural defects introduced during synthesis. Additionally, at 200 °C, mesoporous UiO‐66‐NH₂ retains 96% of its initial weight, compared to 82% for the microporous version. This difference is attributed to the removal of residual solvents such as DMF and water, as well as weakly bound ligands. The data further suggest that approximately 14 wt.% of the mesoporous sample consists of the CAPB surfactant, which decomposes at a higher temperature range.^[^ ^54^, ^55^ ^]^

**Table 1:** BET specific surface area (m^2^ g^−1^_powder_), synthesis temperatures, solvent, total porosity (cm^3^ g^−1^_powder_), microporosity (%), and mesoporosity (%) of the mesoporous versus microporous UiO‐66‐NH_2_.

#	Mesoporous UiO‐66‐NH_2_	Microporous UiO‐66‐NH_2_
Synthesis temperature (°C)	60	90
Solvent	Water	DMF
BET specific surface area (m^2^ g^−1^_powder_)	1000±100	1250±100
Total porosity (cm^3^ g^−1^_powder_)	0.74±0.05	0.51±0.05
Microporosity up to 10 Å	≈39±5%	≈47±2%
Microporosity from 10 to 20 Å	≈13±5%	≈51±2%
Mesoporosity from 20 to 200 Å	≈48±5%	≈1±1%

Table 1 presents data comparing the synthesis processes and properties of microporous and mesoporous UiO‐66‐NH_2_ in powder form. This includes information on synthesis temperatures, solvents used, BET specific surface areas, total porosity (cm^3^ g^−1^), as well as the breakdown of microporosity and mesoporosity percentages. Further details on these properties are provided in the Supporting Information. The BET specific surface area of mesoporous UiO‐66‐NH₂ (1000 ± 100 m^2^ g^−1^_powder_) is lower than its microporous counterpart (1250 ± 100 m^2^ g^−1^_powder_), consistent with previous findings,^[^ ^56^ ^]^ suggesting potential pore blockage by residual CAPB or a more defective framework due to missing linkers. Additionally, when synthesized with acetic acid, the mesoporous UiO‐66‐NH₂ achieves a specific surface area of 1000 ± 100 m^2^ g^−1^_powder_, whereas a higher value of 1410 m^2^ g^−1^_powder_ has been reported for samples prepared using formic acid, highlighting the impact of synthesis conditions on porosity.^[^ ^50^ ^]^ The choice of acetic acid was based on its lower corrosiveness and toxicity, as well as its better environmental compatibility.^[^ ^57^ ^]^ This choice resulted in slightly lower solution acidity compared to formic acid, which may have influenced the crystallization rate and contributed to a slight reduction in surface area. Additionally, Table 1 highlights the notably higher total porosity and mesoporosity % in mesoporous UiO‐66‐NH_2_ compared to its microporous counterpart. The higher total porosity (0.74 cm^3^ g^−1^ vs 0.51 cm^3^ g^−1^) and increase in mesoporosity (48% vs 1%) confirm the role of CAPB in templating mesopores. Finally, the synthesis temperature and solvent used suggest that the synthesis conditions for mesoporous UiO‐66‐NH_2_ are more environmentally friendly, and gentle compared to the evaluated microporous UiO‐66‐NH_2_.

### 2.2 PAN/PVP NFAs

**Figure 2:**
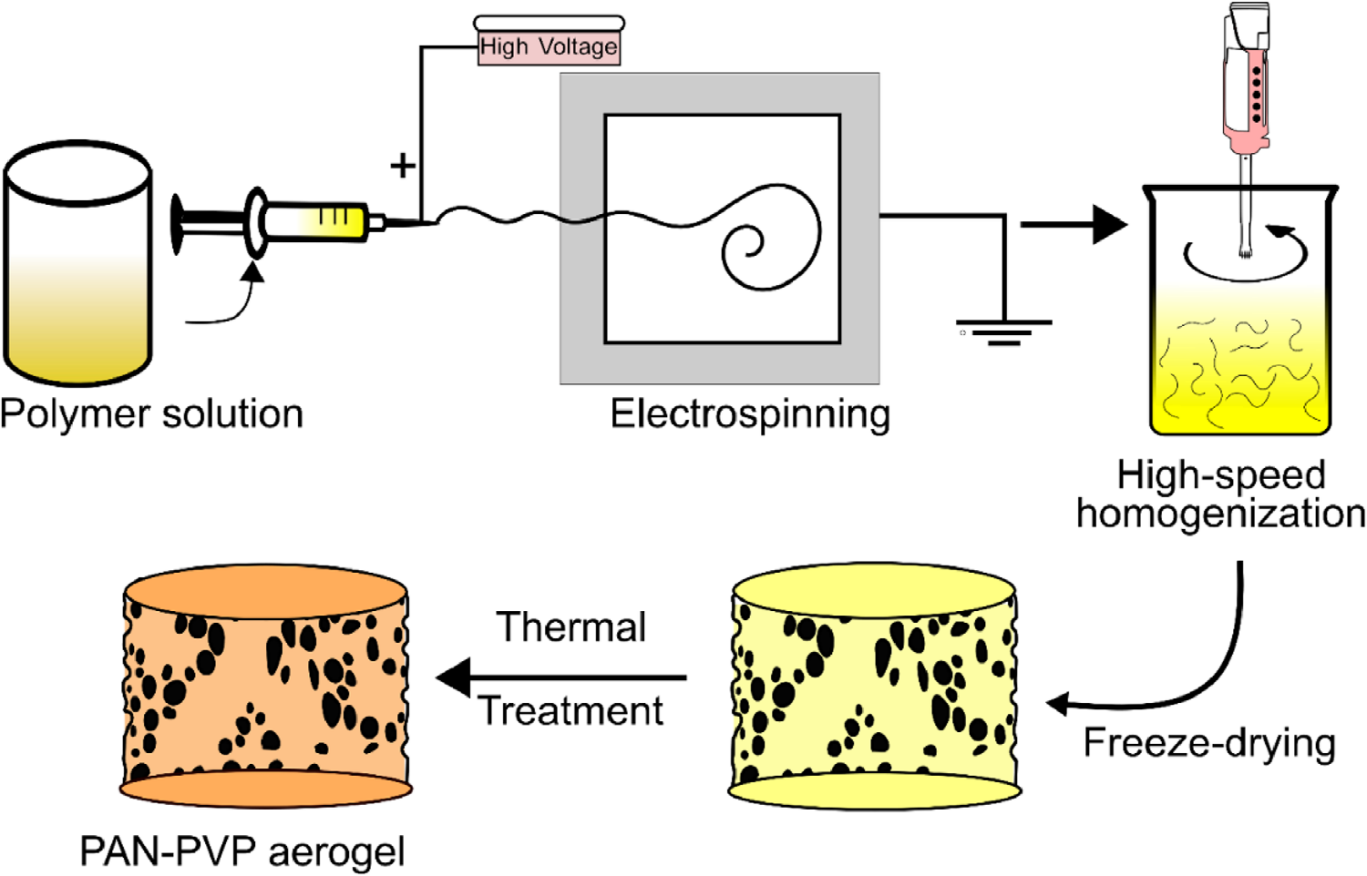
Schematic illustration of the steps involved in the fabrication of the 3D PAN/PVP NFA substrate.

Moving to the substrate, PAN/PVP was used as the material of choice because of its known structural integrity.^[^ ^31^ ^]^ **Figure** **2** illustrates the steps involved in the fabrication of the 3D substrate. The first step involves electrospinning PAN–PVP polymer solution to form defect‐free nanofiber mats using a high‐voltage setup. These nanofiber mats are collected, cut into smaller pieces, and subjected to high‐speed homogenization in a non‐solvent to form a uniform dispersion of nanofibers. The dispersion is subjected to freeze‐drying to sublimate the frozen solvent, following a well‐defined aerogel fabrication procedure, as detailed in the experimental section. The resulting NFAs undergo a series of carefully controlled thermal treatments to form a mechanically robust matrix, which serves as the foundation for subsequent synthesis steps. An important parameter in nanofibrous aerogel formation is the aspect ratio of the dispersion. This is controlled by both the electrospinning and homogenization steps. Previous studies have examined the optimal conditions needed to achieve such properties.^[^ ^9^, ^31^, ^32^ ^]^

**Figure 3:**
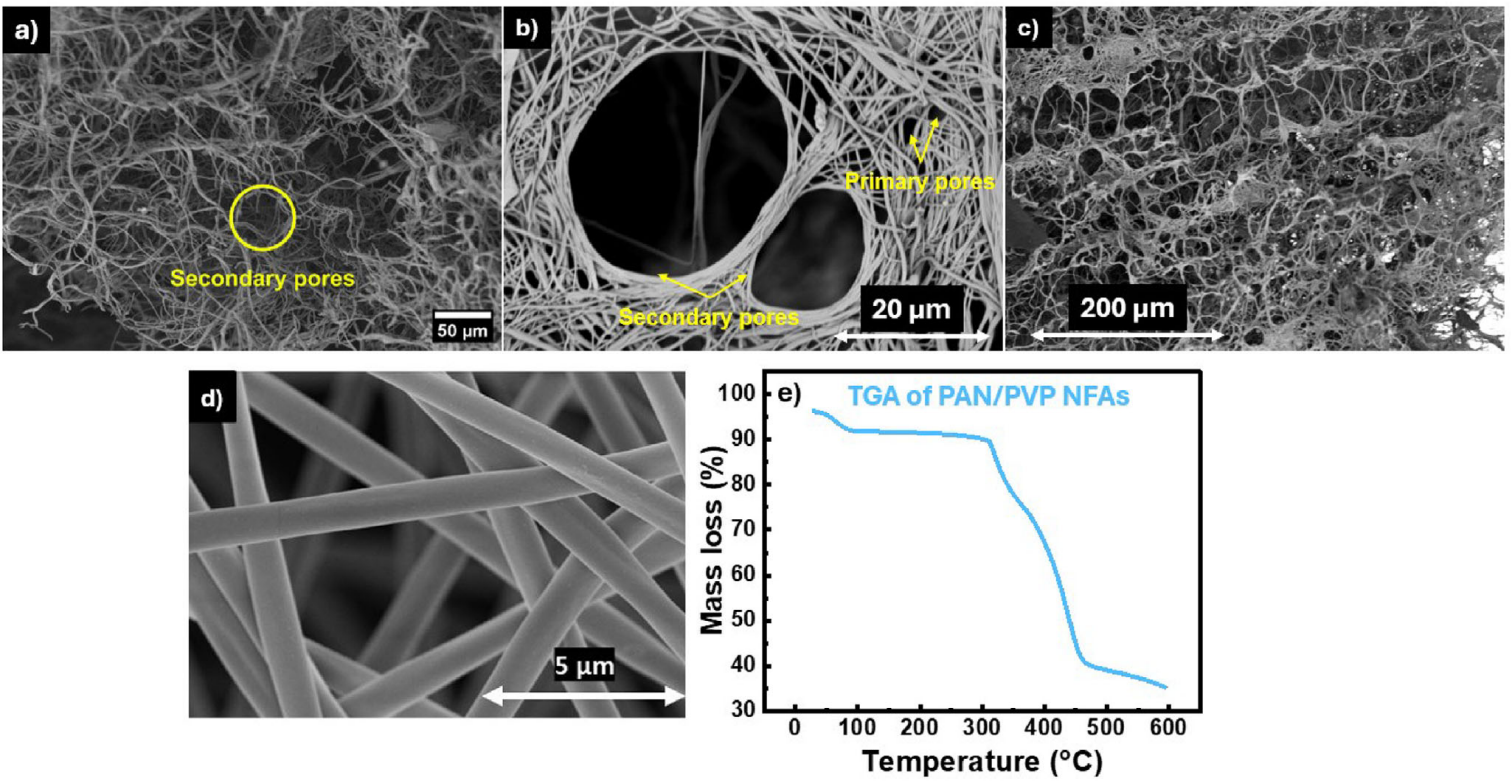
a–d) SEM images of PAN/PVP NFAs; and e) TGA of PAN/PVP NFAs.

**Figure** **3** illustrates the results of SEM, and TGA analyses conducted on the PAN/PVP NFAs. Figure 3a–c highlights the characterization of the developed aerogels post‐thermal treatment, showing SEM images of the NFA consisting of primary and secondary pores. The primary pores (≈1–2 µm) result from the entanglement of fibers, while the secondary pores (≈10–50 µm) are formed after the frozen solvent sublimates as labeled in Figure 3a,b. This structure exhibits an impressive hierarchical porosity of 96.5% as shown in Figure 3c. We want to highlight here that the hierarchical porosity refers specifically to the intrinsic multiscale pore structure of the PAN/PVP nanofibrous aerogel support and not the fibers themselves, which are not porous. SEM offers the best technique to probe such structures.^[^ ^31^, ^32^ ^]^ Notably, the aerogel was formed without multiple solvent exchange steps, demonstrating the ease of forming an interconnected network from the nanofiber dispersion. Figure 3d shows the electrospun nanofibers, which are free of beads or defects, with an average diameter ranging between 0.7 and 1.1 µm, as measured using ImageJ software. Figure 3e presents the thermal decomposition profile of the NFA as it is heated from room temperature to 600 °C. The degradation occurs in two stages: cyclization and dehydrogenation of PAN around 300 °C, followed by the breakdown of PVP organic chains at around 360 °C.^[^ ^31^ ^]^

### 2.3 Mesoporous UiO‐66‐NH_2_ on PAN/PVP NFAs Composites

**Figure 4:**
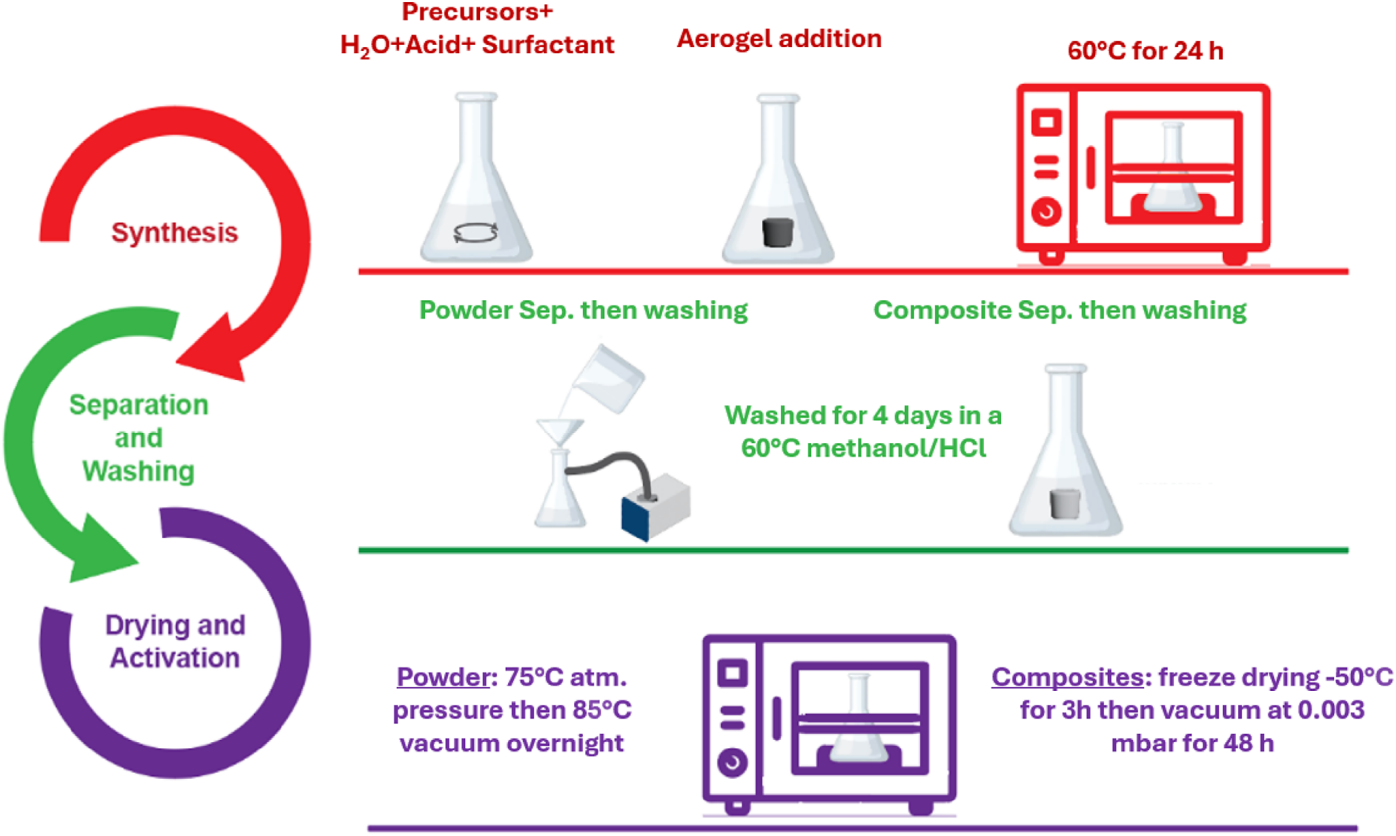
The solvothermal synthesis of the mesoporous UiO‐66‐NH_2_ on nanofibrous aerogels.

We have successfully established the preparation of mesoporous UiO‐66‐NH_2_, which holds great promise for various applications. By incorporating this material into an aerogel matrix, we create a stable and robust platform for its deployment. The aerogel not only enhances the structural integrity and form factor of the MOF but also broadens its potential uses in diverse fields due to its versatility and durability. Therefore, the same MOF was also synthesized as MOF‐PAN/PVP nanofibrous aerogel composites. In this regard, MOF‐aerogels were fabricated by growing MOF on both untreated and treated PAN/PVP aerogels. Treated aerogels are coated with a 20 nm thick TiO_2_ layer via atomic layer deposition (ALD). TiO₂ enhances MOF growth by providing nucleation sites and improving surface properties for better interaction with MOF precursors.^[^ ^47^ ^]^ The TiO₂ layer modifies surface chemistry, enabling uniform and dense MOF films, while also improving adhesion and preventing peeling.^[^ ^56^ ^]^ Mesoporous UiO‐66‐NH_2_ was successfully synthesized solvothermally on both untreated and treated aerogels using 2 different concentrations of the precursors 1X and 2X. An amphoteric surfactant (CAPB) was dissolved in deionized water along with the modulator (acetic acid) and stirred to achieve a homogeneous solution. The precursors were then added to this mixture, followed by stirring. The aerogel was subsequently introduced into the mixture and sonicated to ensure thorough penetration of the suspension into the aerogel. The mixture was heated at 60 °C for 24 h under static conditions. The resulting powder and composite were filtered and washed three times with water to remove the surfactant and twice with methanol to remove any unreacted reactants, then soaked in methanol/HCl mixture (24:1 v/v) for 4 d with the soaking solution replaced every 24 h. The powder was dried at 75 °C overnight and then activated at 85 °C in a vacuum oven overnight while the resulting composite was kept submerged and washed with 1 L of deionized water to move the surfactant, followed by soaking in water for one day and then freeze‐dried at <50 Pa and −50 °C for 48 h as depicted in **Figure** **4**.

**Figure 5:**
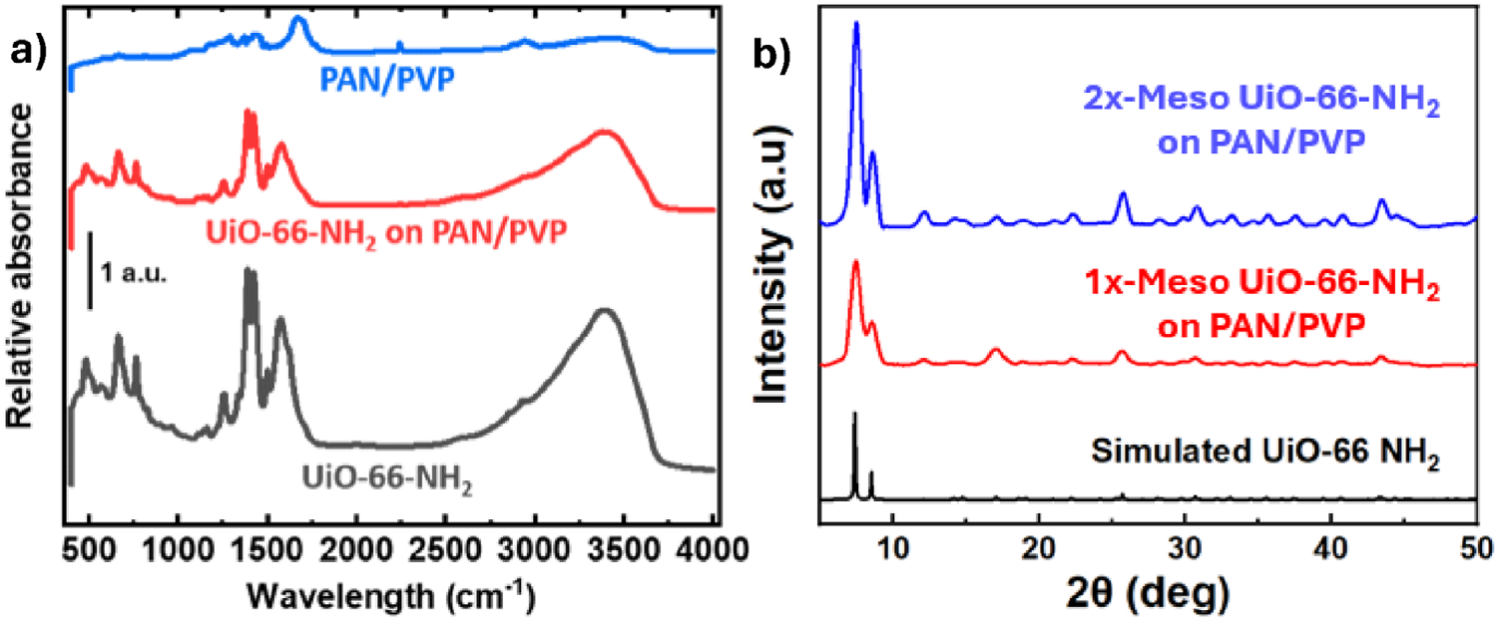
The a) FTIR and b) XRD analyses of the mesoporous UiO‐66‐NH_2_ powder, PAN/PVP NFAs, and mesoporous UiO‐66‐NH_2_ on nanofibrous aerogels.

**Figure** **5** presents FTIR and XRD analyses of mesoporous UiO‐66‐NH_2_ powder, PAN/PVP NFAs, and mesoporous UiO‐66‐NH_2_ on the PAN/PVP NFAs. In Figure 5a, the FTIR spectrum of the thermally treated PAN–PVP NFA displays characteristic absorption peaks at 2245 cm⁻¹, 1665 cm⁻¹, and 1290 cm⁻¹, which correspond to the C≡N group in PAN, and the C═O and C─N groups in PVP. Additionally, the presence of peaks at 1585 cm⁻¹, attributed to C═N and C═C stretching, is due to partial cyclization of the linear nitrile groups in PAN. The FTIR spectrum of UiO‐66‐NH_2_ typically shows characteristic peaks related to both the Zr─O clusters and the organic linker (aminoterephthalic acid). The broad peak around 3300–3500 cm⁻¹ corresponds to the N─H stretching vibrations from the ─NH_2_ group attached to the benzene ring in the aminoterephthalic acid linker. The sharp peak at 1600–1660 cm⁻¹ is due to the C═O stretching vibration in the carboxylate groups (─COOH) of the linker coordinated to the Zr_6_ clusters. The amine bending vibrations at 1500–1600 cm⁻¹ are associated with the ─NH_2_ bending modes. The peaks at 1400–1500 cm⁻¹ are related to C═C stretching vibrations from the aromatic ring of the aminoterephthalic acid linker. The lower‐wavenumber peaks at 500–700 cm⁻¹ correspond to Zr─O stretching modes in the Zr_6_O_4_(OH)_4_ clusters. The successful growth of UiO‐66‐NH_2_ on aerogels is confirmed through FTIR and XRD analyses. FTIR spectra display characteristic peaks of UiO‐66‐NH_2_ on the PAN/PVP matrix, indicating that no reactions occurred between the precursors and the PAN/PVP during synthesis. Additionally, the XRD patterns align with the simulated spectra, confirming the proper integration of the crystalline MOF onto the aerogel scaffold. In Figure 5b, the intensities of UiO‐66‐NH_2_ increase as the precursor concentration rises from 1X to 2X, indicating the formation of more crystalline structures. This is because a higher concentration of 2X precursors provides more building blocks for MOF crystal formation than 1X, leading to improved crystallinity and more well‐defined structures as the synthesis process progresses. Figure S1 (Supporting Information) displays the TGA plots for the composite NFA with 1X and 2X MOF concentrations follow similar trends as the individual components, showing PAN cyclization and PVP decomposition around 400 °C.^[^ ^31^ ^]^

**Figure 6:**
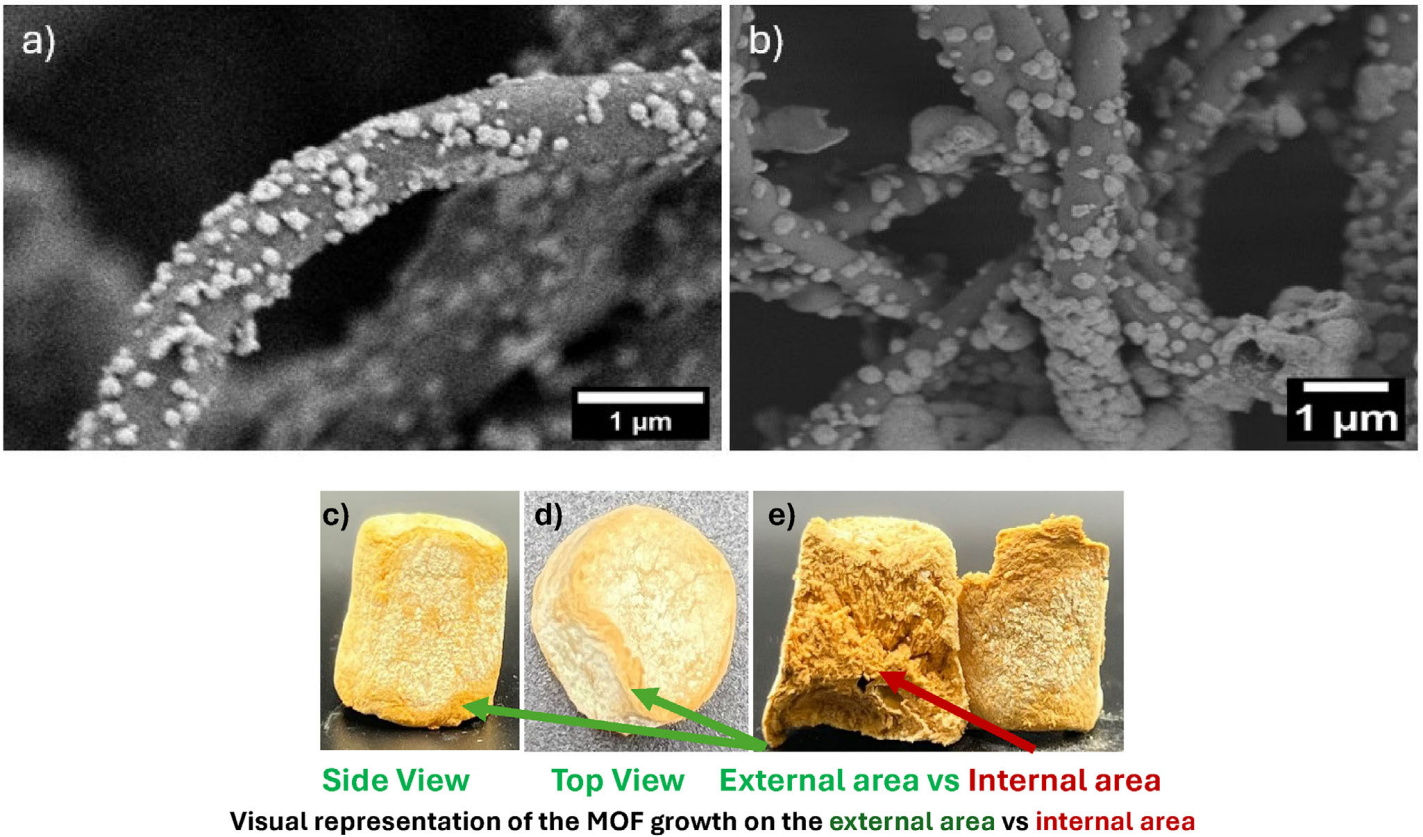
SEM images of a) 2X‐mesoporous UiO‐66‐NH_2_‐PAN/PVP; b) 1X‐mesoporous UiO‐66‐NH_2_‐PAN/PVP aerogel composites; c) Side view of 1X‐mesoporous UiO‐66‐NH_2_‐PAN/PVP aerogel; d) Top view of 1X‐mesoporous UiO‐66‐NH_2_‐PAN/PVP aerogel; e) Comparison between MOF formation on the internal area versus the external area of the 1X‐mesoporous UiO‐66‐NH₂‐PAN/PVP aerogel (≈450 m^2^ g^−1^_comp_).

**Figure** **6**a,b show SEM images of 2X and 1X mesoporous UiO‐66‐NH_2_ on NFA, respectively. In both concentrations, the solvothermally grown MOF was uniformly distributed across the nanofibers, consistent with the BET specific surface area and the high MOF loading results. Additional SEM images at various magnifications, presented in the section as Figures S2 and S3 (Supporting Information), further corroborate the homogeneous distribution and significant MOF loading throughout the nanofibers. **Table** **2** presents data on BET speific surface area, MOF loading determined from BET analysis, and adhesion testing results. The highest surface area was observed for 2X‐mesoporous UiO‐66‐NH_2_ on PAN/PVP which is almost twice the highest previously reported in the literature,^[^ ^41^ ^]^ followed by 2X‐mesoporous UiO‐66‐NH_2_ on PAN‐PVP@TiO_2_. In the 1X‐mesoporous UiO‐66‐NH₂‐PAN/PVP aerogel, a specific surface area gradient appears in MOF growth, with higher surface growth on the external area (more than 400 m^2^ g^−1^_comp_) compared to the aerogel internal area (less than 200 m^2^ g^−1^_comp_). This gradient is not observed in the 2X concentration sample. We hypothesize that the lower concentration in the 1X sample does not create a sufficient driving force for the MOF precursors to diffuse to the aerogel's center as effectively as in the 2X sample as shown in Figure 6c–e. Notably, the synthesis of mesoporous material at lower temperatures enabled successful solvothermal growth of mesoporous UiO‐66‐NH_2_ on untreated and treated aerogels while preserving mechanical integrity. Adhesion of the MOF to the substrate, evaluated through manual compression and agitation, brushing, and exposure to a pressurized air stream, is outlined in Table 2. Remarkably, despite the lower synthesis temperature and change in solvent, excellent adhesion of mesoporous UiO‐66‐NH_2_ was observed on all substrates with both concentrations as illustrated and further detailed in Figure 9d. Normally, higher synthesis temperatures can enhance the adhesion of MOFs on fibers by improving crystallinity and increasing the nucleation rate, which provides more contact points between the MOF and the fiber surface. However, elevated temperatures may also pose risks, such as potential degradation of the fibers. The choice of organic solvents significantly affects adhesion as well; solvents can enhance solubility and precursor interaction, improve surface wettability, and facilitate favorable intermolecular interactions. Additionally, the volatility of the solvent influences the drying rate during synthesis, which can impact the uniformity and quality of the MOF layer. Therefore, careful selection of both synthesis temperature and solvent is essential for optimizing MOF adhesion on fibers for various applications. We investigated previously the growth of microporous UiO‐66‐NH₂ using DMF at 90 °C and mesoporous UiO‐66‐NH₂ synthesized in water at 60 °C on PP substrates coated with a 20 nm TiO₂ layer.^[^ ^47^ ^]^ The mesoporous MOF exhibited a threefold higher surface area and formed a more uniform thin film, whereas the microporous counterpart showed less cohesive growth, as confirmed by SEM images in Figure S4 (Supporting Information). These results underscore the crucial influence of solvent and temperature on MOF adhesion while keeping other synthesis parameters unchanged. The use of lower temperatures and water as a solvent, combined with the amphoteric surfactant CAPB, significantly improves MOF adhesion and fiber integrity. CAPB enhances fiber wettability, promotes electrostatic interactions with MOF precursors, and removes surface contaminants, ensuring uniform growth. Additionally, lower synthesis temperatures minimize thermal stress, preserving the fibers’ mechanical properties while supporting strong MOF attachment, making the composites suitable for various applications.^[^ ^47^ ^]^

**Table 2:** BET specific surface area results of uncoated and coated 1X‐mesoporous UiO‐66‐NH_2_‐PAN/PVP and 2X‐mesoporous UiO‐66‐NH_2_‐PAN/PVP aerogel composites with average MOF loading from BET (%) and adhesion.

#	BET specific surface area [m^2^ g^−1^_comp_] Internal	BET specific surface area [m^2^ g^−1^_comp_] External	Average MOF loading from BET [%]	Adhesion
1X‐mesoporous UiO‐66‐NH_2_ on PAN‐PVP	100	430	28%	Excellent
1X‐mesoporous UiO‐66‐NH_2_ on PAN‐PVP@TiO_2_	180	460	33%	Excellent
2X‐mesoporous UiO‐66‐NH_2_ on PAN‐PVP	910	830	73%	Excellent
2X‐mesoporous UiO‐66‐NH_2_ on PAN‐PVP@TiO_2_	820	790	69%	Excellent

**Figure 7:**
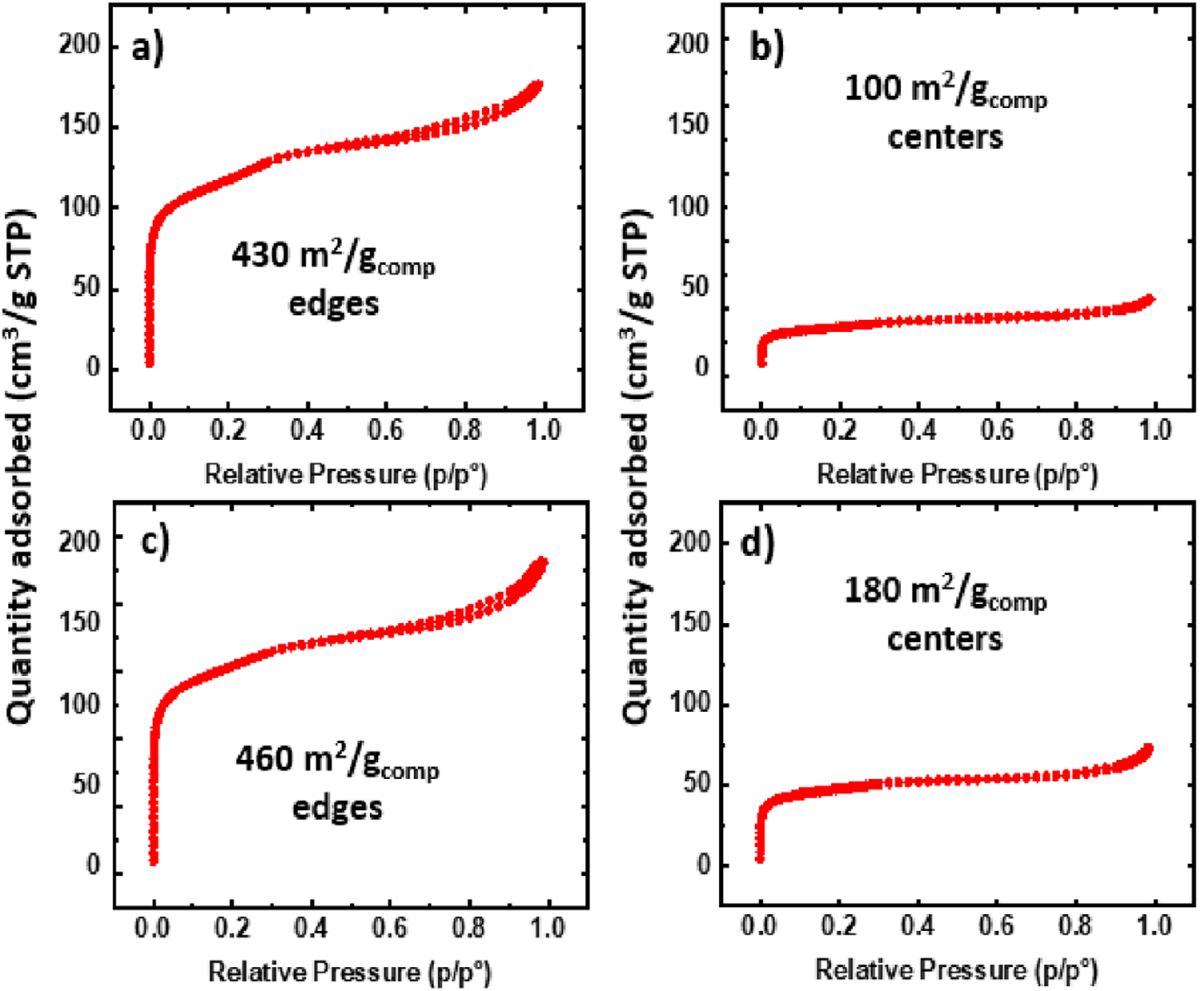
N_2_ isotherms of 1X‐mesoporous UiO‐66‐NH_2_‐PAN/PVP aerogel composites a,b) untreated substrate c,d) treated substrate with 20 nm TiO_2_.

**Figure 8:**
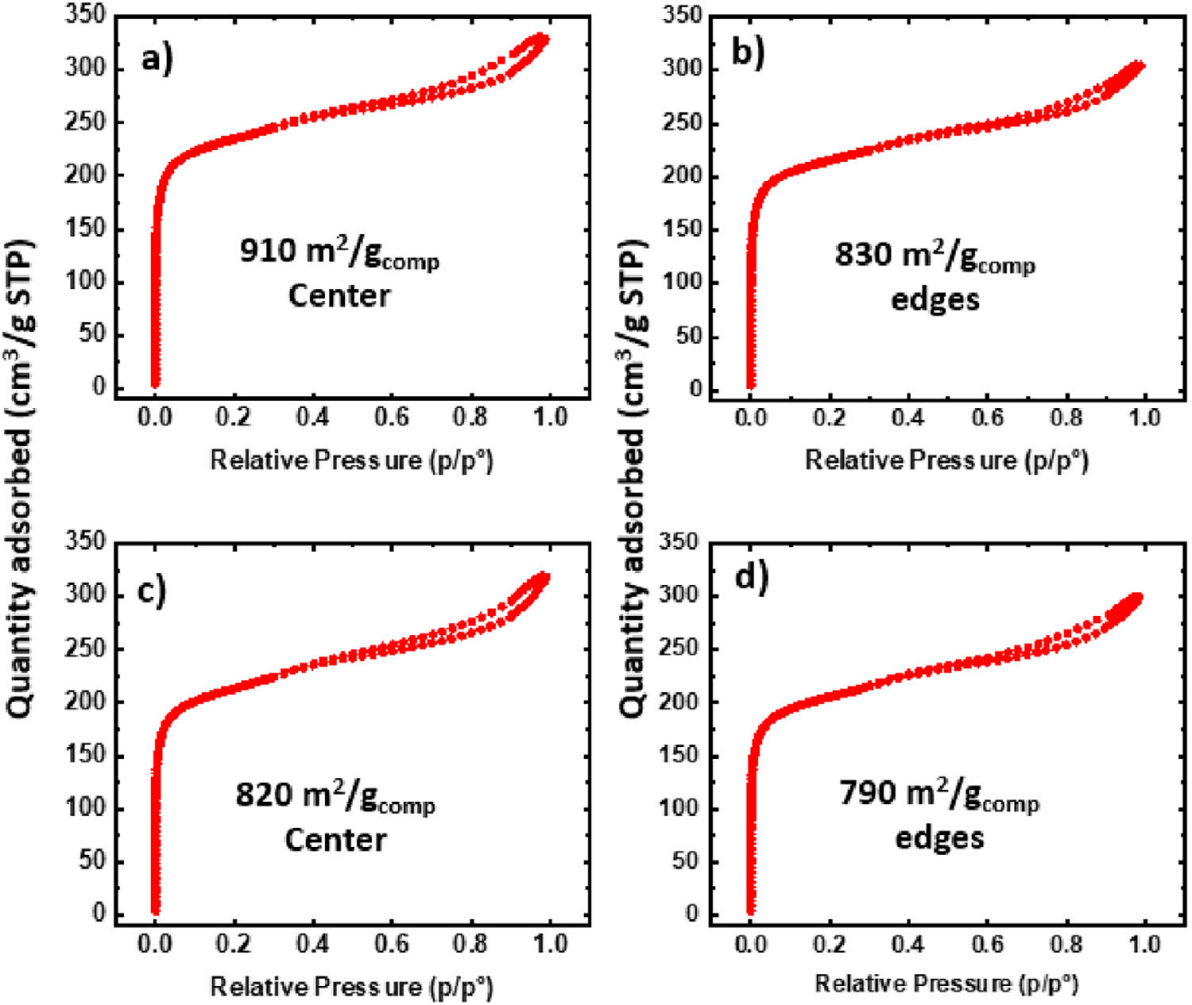
N_2_ isotherms of 2X‐mesoporous UiO‐66‐NH_2_‐PAN/PVP aerogel composites a,b) untreated substrate c,d) treated substrate with 20 nm TiO_2_.

**Figures** **7** and **8** illustrate the isotherms for all mesoporous UiO‐66‐NH_2_/NFA composites. Each isotherm exhibits a Type I and Type IV blend, indicating that the aerogels' MOF crystals retain microporosity and mesoporosity. Notably, the 2X‐mesoporous UiO‐66‐NH_2_‐PAN/PVP@TiO_2_ center demonstrates the highest mesoporosity, boasting a total porosity of 0.48 cm^3^ g^−1^_comp_, with 36% mesoporosity. This results in the highest mesoporous pore volume, reaching up to 0.173 cm^3^ g^−1^_comp_. The total pore volume, mesoporosity, and mesoporosity % for all MOF‐NFA composites are detailed in **Table** **3**. This further strengthens the argument for the preservation of the MOF's intrinsic porous structure on the aerogel matrix without any compromise. As discussed, prior attempts to directly integrate mesoporous MOFs onto fabrics have faced challenges due to either pore blockage or degradation of the MOF structure.^[^ ^23^ ^]^

**Table 3:** Total pore volume (cm^3^ g^−1^_comp_), mesoporosity (%), and total mesopore volume of all the mesoporous UiO‐66‐NH_2_/nanofibrous PAN/PVP aerogels.

MOF composites	Total pore volume (cm^3^ g^−1^_comp_)	Mesoporosity [% (>20 Å up to 200 Å)	Total mesopore volume (cm^3^ g^−1^_comp_)
1X‐mesoporous UiO‐66‐NH_2_‐PAN/PVP center	≈0.064	≈47%	0.03
1X‐mesoporous UiO‐66‐NH_2_‐PAN/PVP edge	≈0.27	≈41%	0.11
1X‐mesoporous UiO‐66‐NH_2_‐PAN/PVP@TiO_2_ center	≈0.11	≈37%	0.041
1X‐mesoporous UiO‐66‐NH_2_‐PAN/PVP@TiO_2_ edge	≈0.28	≈40%	0.112
2X‐mesoporous UiO‐66‐NH_2_‐PAN/PVP center	≈0.49	≈31%	0.1519
2X‐mesoporous UiO‐66‐NH_2_‐PAN/PVP edge	≈0.45	≈31%	0.1395
2X‐mesoporous UiO‐66‐NH_2_‐PAN/PVP@TiO_2_ center	≈0.48	≈36%	0.1728
2X‐mesoporous UiO‐66‐NH_2_‐PAN/PVP@TiO_2_ edge	≈0.45	≈34%	0.153

**Figure 9:**
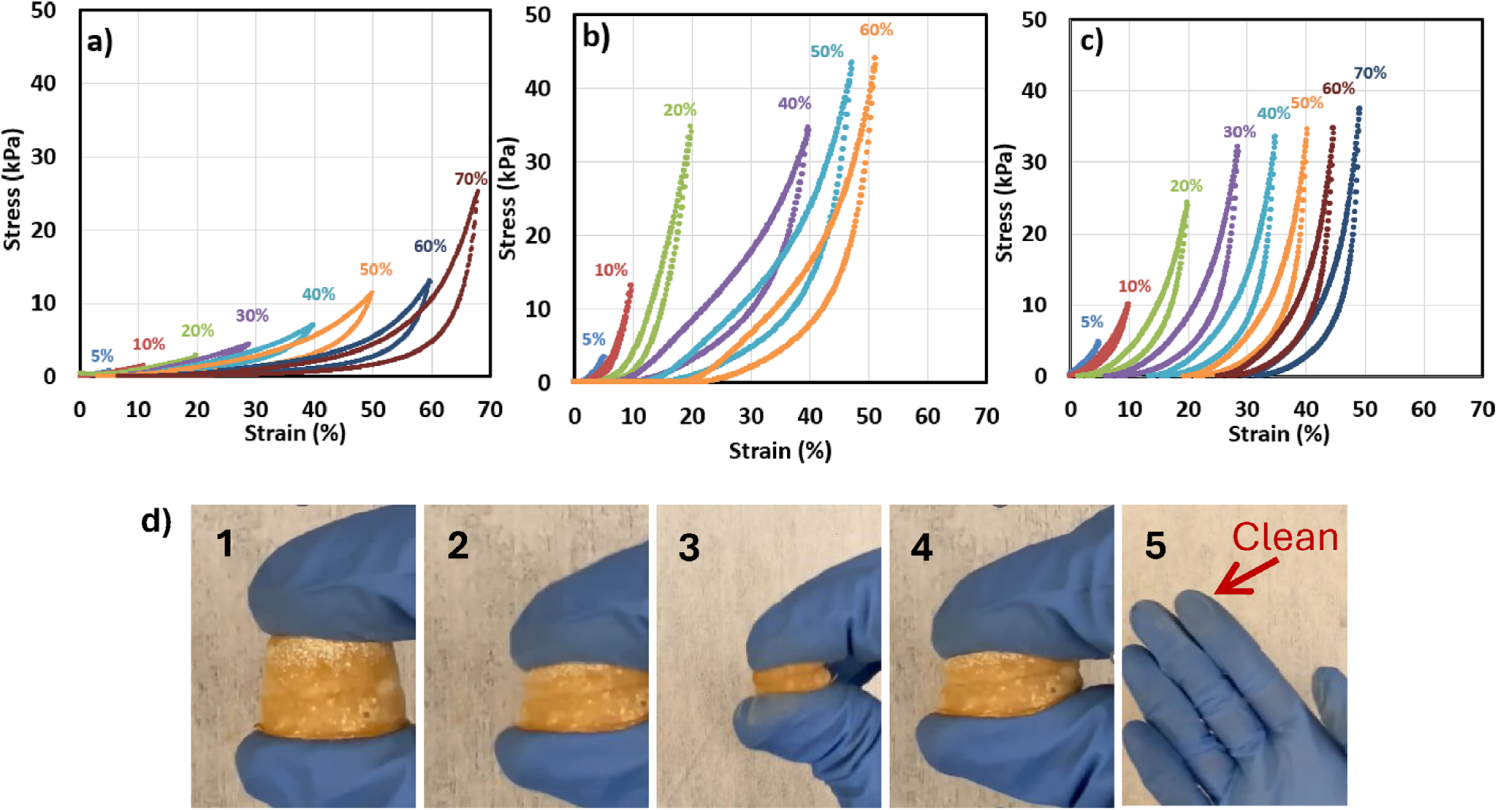
Dynamic mechanical analysis: a) PAN/PVP; b) 1X‐mesoporous UiO‐66‐NH_2_ on PAN/PVP; c) 2X‐Mesoporous UiO‐66‐NH_2_ on PAN/PVP; d) Manual cyclic compression of the 2X‐Mesoporous UiO‐66‐NH_2_ on PAN/PVP (900 m^2^ g^−1^_comp_ almost 80% MOF loading).

**Figure 10:**
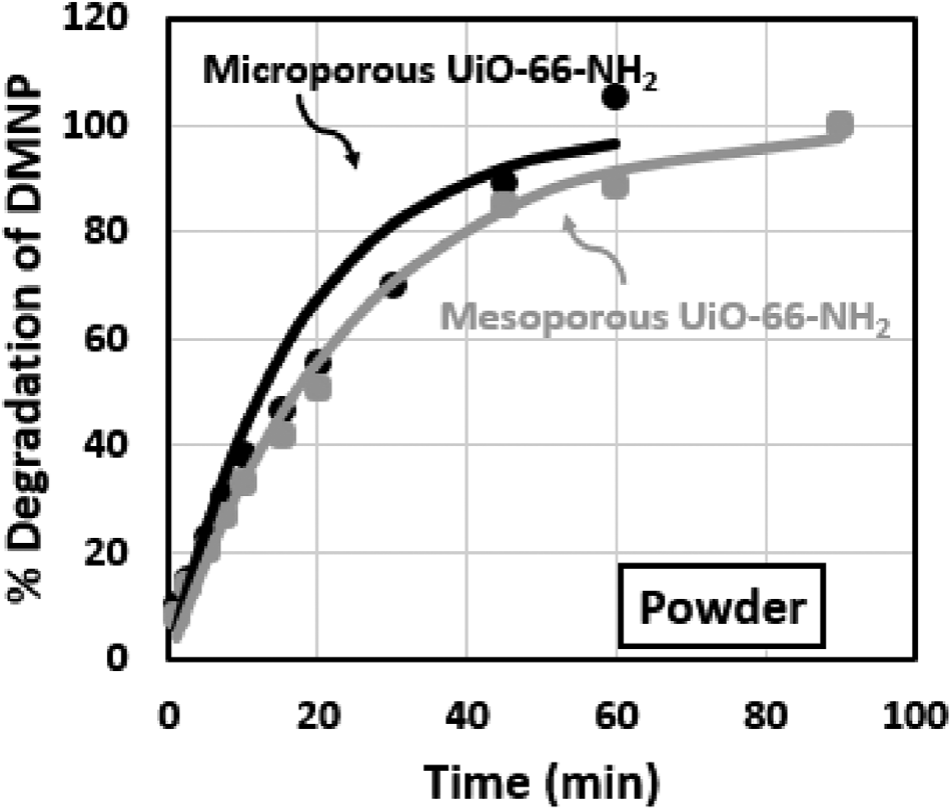
Pseudo first‐order kinetic model fitting the experimental data of % decomposition of DMNP versus Time of mesoporous UiO‐66‐NH_2_ and microporous UiO‐66‐NH_2_ powders.

The prime benefit of an aerogel matrix lies in its mechanical robustness and the ability to withstand multiple compressions without losing its structural integrity. NFAs exhibit distinct mechanical properties, setting them apart from conventional aerogels, which often lack an interconnected network. Conventional aerogels are commonly fabricated by removing the liquid phase from a gel via freeze‐drying or supercritical drying. These processes often lead to the formation of “necks” within the structure, compromising the aerogel's mechanical properties.^[^ ^58^ ^]^ Additionally, the multi‐step process of solvent extraction and drying is labor‐intensive and inefficient.^[^ ^33^, ^34^ ^]^ Aerogels produced through these methods lack an interconnected network, resulting in brittleness and insufficient hierarchical porosity, which limits their structural integrity and functional versatility.^[^ ^36^, ^37^ ^]^ Several studies have demonstrated that incorporating a fibrous network in the aerogel matrix reduces the formation of these “necks,” thereby leading to a stronger and robust aerogel.^[^ ^9^, ^31^, ^59^ ^]^ NFAs are highly porous structures composed of a dense network of nanofibers, enabling efficient dissipation of applied stress. To evaluate their mechanical robustness and compressibility, cyclic compressive experiments were conducted on pristine PAN/PVP NFAs, along with 1X and 2X mesoporous UiO‐66‐NH_2_ on PAN/PVP NFAs as illustrated in Figure 10. In cyclic stress‐strain tests, the slope of the hysteresis loops reflects the material's stiffness or elastic modulus, providing insight into its deformation response and recovery. A steeper slope in the hysteresis loop generally indicates higher stiffness, meaning the material can resist deformation and return to its original form more effectively after being loaded and unloaded. In contrast, a shallower slope suggests lower stiffness, associated with more flexible materials that experience greater deformation under similar stress.^[^ ^32^ ^]^ The slope can also offer information about energy dissipation; a loop with a consistent slope over cycles indicates strong fatigue resistance and minimal permanent deformation, important for materials that must withstand repetitive stress.^[^ ^60^ ^]^ The compression‐relaxation curves obtained from these tests reveal three characteristic deformation regimes. At low strains, the NFAs exhibit a Hookean or linear elastic behavior.^[^ ^61^ ^]^ As the strain increases, the material enters a plateau region, where elastic buckling of the cell walls occurs. At higher strains, a densification regime emerges, characterized by a steep increase in stress (σ).^[^ ^62^ ^]^ Most of the loading‐unloading curves show strain (ε)‐dependent hysteresis loops, indicating that energy is dissipated within the fibrous network. This energy dissipation suggests that the fibrous structure helps absorb and release energy during compression and recovery.^[^ ^60^ ^]^ Notably, the incorporation of UiO‐66‐NH_2_ within the NFAs does not significantly alter the mechanical performance of the structure. Dynamic mechanical analysis (DMA) further supports these findings, providing insights into the elastic behavior and shape retention of the materials under strain. The pristine PAN/PVP NFAs remain almost elastic up to 47% strain, with an overall shape loss of 16.8% as shown in Figure S5a (Supporting Information). In contrast, the 1X‐mesoporous UiO‐66‐NH_2_ loaded NFAs exhibit improved elasticity, sustaining nearly elastic behavior up to 50% strain with a reduced shape loss of 14.5% as shown in Figure S5b (Supporting Information). A declining slope over successive cycles was observed for the 1x‐mesoporous UiO‐66‐NH₂‐loaded NFAs, potentially indicating damage accumulation or bonding issues. Remarkably, the 2X‐mesoporous UiO‐66‐NH_2_ NFAs demonstrate the highest mechanical resilience, maintaining almost elastic behavior up to 70% strain and experiencing only 6% shape loss as shown in Figure S5c (Supporting Information). The MOF‐loaded NFAs demonstrate excellent shape recovery and compressibility, even under high strains. This ability to maintain structural integrity is attributed to the retention of the hierarchical porous framework and the formation of intermolecular crosslinking between the PAN and PVP. Additionally, the growth of MOFs on the fibers provides additional support, enabling the fibers to withstand higher stress under the same strain. These structural features play a critical role in preserving the mechanical strength and recovery behavior of the NFAs, making them highly suitable for applications requiring robust, lightweight materials. Manual cyclic compression testing was conducted on the 2X‐Mesoporous UiO‐66‐NH₂ on PAN/PVP composite to evaluate MOF adhesion on this high‐loading (80% MOF) sample with a BET specific surface area of 900 m^2^ g^−1^. As shown in **Figure** **9**d, after several compression cycles, no MOF residue was visible on the gloves, highlighting the strong binding of the MOF on the NFA structure.

Compared to the PAN/PVP‐based aerogels, which demonstrated superior mechanical stability and high surface area, the solvothermal growth of mesoporous UiO‐66‐NH₂ on CDA/silica aerogels resulted in composites with significant mechanical challenges. As shown in Table S2 (Supporting Information), these composites initially achieved high specific surface areas of approximately 300 ± 60 m^2^ g^−1^ at 1X precursor concentrations; however, Figure S7e (Supporting Information) illustrates that they became brittle with diminished mechanical resilience. Reducing precursor concentrations halved the specific surface area to around 60 ± 5 m^2^ g^−1^, but the brittleness persisted. Isotherm analysis Figure S7a–d (Supporting Information) confirmed a combination of micropores and mesopores (Type I and Type IV characteristics). To improve mechanical stability, an alternative freeze‐drying method was employed, resulting in composites with a specific surface area of approximately 170 m^2^ g^−1^ Table S3 (Supporting Information). However, despite maintaining a microporous and mesoporous structure (Figure S8, Supporting Information) the freeze‐dried composites suffered from structural softness and collapse, further limiting their mechanical robustness.

### 2.4 DMNP Detoxification

After analyzing MOF powders and MOF aerogel composites, our focus shifted to evaluating their effectiveness in degrading OP compounds. We measured the hydrolysis rate of methyl paraoxon (DMNP, GD simulant) for MOF powders and composites, alongside conducting live agent degradation tests using both concentrations of mesoporous UiO‐66‐NH_2_ aerogel composites.

Results from the analysis of MOF powders are summarized in **Table** **4** and **Figure** **10**, where the lines are fit to a pseudo‐first‐order reaction equation while the dots represent experimental data. Microporous and mesoporous UiO‐66‐NH_2_ samples were tested for DMNP hydrolysis in a buffered solution (pH = 10) at room temperature, using 2.5 mg of MOF powder. The microporous UiO‐66‐NH_2_ demonstrated a half‐life (*t*_1/2_) of 15 min, whereas the mesoporous variant showed a *t*_1/2_ of 20 min. Comparing the degradation rate of DMNP in mesoporous UiO‐66‐NH_2_ and microporous UiO‐66‐NH_2_, the microporous UiO‐66‐NH_2_ outperformed the mesoporous one. Despite mesoporous UiO‐66‐NH_2_ having larger pores, the higher surface area of 2X‐microporous UiO‐66‐NH_2_ may compensate for its relatively smaller pore size, resulting in faster kinetics. The rapid degradation of DMNP was attributed not only to the mesopores and high surface area but also to the NH_2_ functional groups in UiO‐66‐NH_2_, which act as nucleophiles enhancing hydrolysis by interacting with the phosphorus atom of the OP compound.^[^ ^63^ ^]^ Additionally, it facilitates bond cleavage by forming a covalent bond with the phosphorus atom, facilitating the cleavage of the P‐O bond, which is crucial for enhancing the kinetics of the hydrolysis process.

**Table 4:** DMNP degradation test results of mesoporous and microporous UiO‐66‐NH_2_ powders.

#	Mesoporous UiO‐66‐NH_2_	2X‐Microporous UiO‐66‐NH_2_
DMNP *t*_1/2_ (min)	20	15
DMNP Complete conversion (min)	90	<60

**Figure 11:**
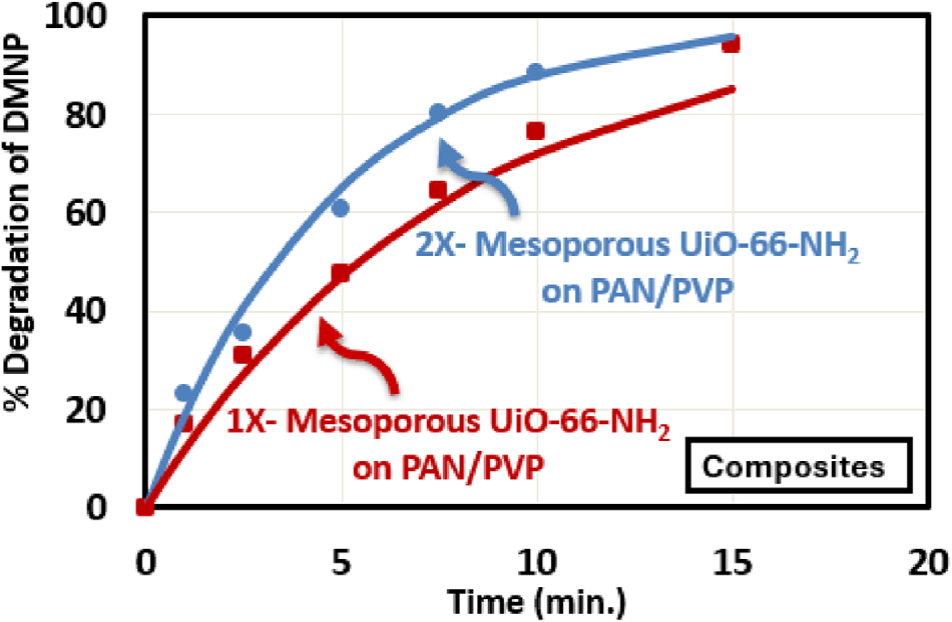
Pseudo first‐order kinetic model fitting the experimental data of % decomposition of DMNP versus Time of 1X‐mesoporous UiO‐66‐NH_2_‐PAN/PVP, and 2X‐mesoporous UiO‐66‐NH_2_‐PAN/PVP aerogel composites.

For MOF‐aerogel composites, OP hydrolysis results are outlined in **Table** **5** and **Figure** **11**, where the lines in Figure 11 are fit to a pseudo‐first‐order reaction equation while the dots represent experimental data. DMNP hydrolysis was conducted in a buffered solution (pH = 10) at room temperature using 14 mg of 1X and 2X mesoporous UiO‐66‐NH_2_ aerogel composites. The 2X‐mesoporous UiO‐66‐NH_2_ composite exhibited a *t*_1/2_ of 3.3 min, achieving complete conversion in 15 min, while the 1X‐mesoporous UiO‐66‐NH_2_ aerogel composite demonstrated a *t*_1/2_ of 5.5 min, and complete conversion in 17 min. The PAN/PVP did not exhibit any conversion even after 1 h of reaction. We avoid comparing the mesoporous UiO‐66‐NH_2_ aerogel with the microporous variant, as the latter requires toxic DMF and high temperatures (90–120 °C), which would likely degrade the composites' mechanical integrity and make them unsuitable for our applications.

**Table 5:** Half‐life time and complete conversion time of DMNP hydrolysis test of mesoporous UiO‐66‐NH_2_ on PP@TiO_2_, 1X‐mesoporous UiO‐66‐NH_2_‐PAN/PVP, and 2X‐mesoporous UiO‐66‐NH_2_‐PAN/PVP aerogel composites.

#	2X‐Mesoporous UiO‐66‐NH_2_ on PAN/PVP	1X‐Mesoporous UiO‐66‐NH_2_ on PAN/PVP	Microporous UiO‐66‐NH_2_ on PP@TiO_2_^[^ ^56^ ^]^	Microporous UiO‐66‐NH_2_ (3) on PP@TiO_2_^[^ ^64^ ^]^
*t*_1/2_ (min)	3.3	5.5	15	5
Complete conversion (min)	15	17	>90	>40

The results presented in Figure 11 were obtained using the same protocol as previously reported for the analysis of microporous UiO‐66‐NH_2_ (3) on PP@TiO_2_, allowing for a control comparison with the results presented here.^[^ ^64^ ^]^ Previously reported values for 3 layers of microporous UiO‐66‐NH_2_ (3) on PP@TiO_2_ control materials show significant differences compared to one layer of the mesoporous UiO‐66‐NH_2_ aerogel composites presented in this study.^[^ ^64^ ^]^ For instance, a specific surface area of 132 m^2^ g^−1^_comp_ was achieved with microporous UiO‐66‐NH_2_ (3) on PP@TiO_2_, delivering a *t*_1/2_ of approximately 5 min and requiring over 40 min for complete conversion.^[^ ^64^ ^]^ Another study reported a single layer of microporous UiO‐66‐NH_2_ on PP@TiO_2_ with a specific surface area of 65 m^2^ g^−1^_comp_, exhibiting a *t*_1/2_ of 15 min and complete conversion in over 90 min.^[^ ^56^ ^]^ These results highlight the substantial improvements in surface area and catalytic efficiency achieved with the mesoporous UiO‐66‐NH_2_ aerogel composites developed in this work. This comparison underscores the substantial impact of utilizing mesoporous MOFs and the high absorption capacity of nanofibrous aerogels on DMNP hydrolysis. Moreover, the study reveals that a single layer of mesoporous UiO‐66‐NH_2_ performed better than three layers of unmodified microporous UiO‐66‐NH_2_ on PP@TiO_2_.^[^ ^64^ ^]^ Table S1 (Supporting Information) compares the performance of this work with other MOF‐based composites, including fabrics, textiles, and hydrogels, in relation to DMNP hydrolysis. The 2X‐mesoporous UiO‐66‐NH_2_ on PAN/PVP demonstrates DMNP hydrolysis comparable to those reported in the literature while also existing in the form of a robust 3D matrix. This further supports the notion that larger pore sizes facilitate the easier transport of bulky DMNP molecules to active sites, thereby circumventing diffusion as a limiting step for the hydrolysis reaction. The combination of a higher surface area and MOF loading enhanced the availability of active sites, resulting in the more rapid degradation of DMNP molecules.

For MOF‐fabric tests, performance evaluation was based on the total mass of the composite rather than solely the MOF content, following established methodologies from previous studies.^[^ ^47^, ^56^, ^65^ ^]^ This approach reflects practical application considerations, ensuring that material performance aligns with real‐world constraints, such as minimizing the weight burden on users. The use of 14 mg in these tests maintains consistency with prior work, ensuring experimental reproducibility while supporting meaningful comparisons.

### 2.5 CWAs Detoxification

Following the simulant hydrolysis tests, solid‐state experiments were conducted to assess the degradation of live chemical agents. **Table** **6** presents the results of degradation experiments for GD and HD using mesoporous UiO‐66‐NH_2_ aerogel composites, including 1X and 2X concentrations. Due to the high toxicity of these live agents, experiments were conducted only once for each sample and reported values were approximations. Nonetheless, the findings revealed that mesoporous UiO‐66‐NH_2_ aerogel composites achieved nearly complete conversion of GD within 24 h, with lower degradation observed for HD. Here we did the CWAs degradation tests on the composites only as GD and HD dosing to UiO‐66‐NH_2_ powders typically achieves 100% conversion within 24 h, facilitated by physical agitation to enhance solid–liquid contact efficiency. In contrast, dosing GD and HD onto MOF‐composites involves spot application at random locations, potentially leading to localized saturation with CWA and leaving some areas unreacted. Capillary action and vapor diffusion work to equilibrate the chemical across the MOF‐composite.

**Table 6:** GD and HD degradation test of 1X and 2X mesoporous UiO‐66‐NH_2_‐PAN/PVP aerogel composites.

#	2X‐Mesoporous UiO‐66‐NH_2_ on PAN/PVP		1X‐Mesoporous UiO‐66‐NH_2_ on PAN/PVP		PAN/PVP
	Non	TiO_2_	Non	TiO_2_	
Degradation of GD in 1h (%)	20	34	17	22	–
Degradation of GD in 4 h (%)	17	48	35	12	–
Degradation of GD in 24 h (%)	83	95	76	56	18
Degradation of HD in 24 h (%)	54	72	43	32	12

The 2X‐mesoporous UiO‐66‐NH_2_ on treated aerogels displayed enhanced degradation of GD compared to microporous UiO‐66‐NH_2_ (3) on treated fabrics.^[^ ^64^ ^]^ Three layers of microporous UiO‐66‐NH_2_ (3) on treated fabrics exhibit a GD half‐life of approximately 5 hours,^[^ ^64^ ^]^ compared to about 4 hours for a single layer of mesoporous UiO‐66‐NH_2_ on treated aerogel, using the same mass for both tests.^[^ ^64^ ^]^ This enhancement is primarily attributed to the presence of mesochannels and mesopores, facilitating the accessibility of bulky compounds like GD to the active sites. Moreover, the elevated surface area resulting from the nanofibrous substrate indicates a greater number of accessible active sites.^[^ ^50^ ^]^

The 2X‐mesoporous UiO‐66‐NH_2_ on PAN/PVP@TiO_2_ demonstrated almost complete degradation of GD within 24 hours, with a *t*_1/2_ of around 4 h. Even the non‐coated 2X‐mesoporous UiO‐66‐NH_2_ on PAN/PVP achieved over 80% degradation in the same period. The 1X‐mesoporous UiO‐66‐NH_2_ on PAN/PVP also showed high GD degradation percentages, albeit lower than the 2X‐mesoporous UiO‐66‐NH_2_ on PAN/PVP. Higher precursor concentrations in MOF synthesis typically increase nucleation rates, resulting in a higher density of MOF crystals. This can enhance the overall yield of the MOF material and potentially improve the crystallinity and structural integrity of the resulting framework.

Regarding HD degradation, both concentrations of mesoporous UiO‐66‐NH_2_ aerogel composites exhibited substantially higher degradation performance compared to mesoporous UiO‐66‐NH_2_ grown on PP@TiO_2_ (around 18%).^[^ ^47^ ^]^ The high degradation of HD for the former composites is attributed to its effective infiltration into the MOF‐nanofibrous aerogel composite, facilitated by the substrate's high porosity exceeding 90%. In contrast, MOF‐fabric composites limit the transport of the agent to the active catalytic sites.^[^ ^47^ ^]^ The infiltration and diffusion of HD through the fabric were limited, likely due to the lower porosity and thicker fiber diameters in the micrometer range, which could adsorb more water in these tests, thus hindering the insoluble HD.

## 3 Conclusion

In summary, the exploration of mesoporous UiO‐66‐NH_2_ integrated into 3D PAN/PVP aerogels represents a significant advancement in the development of materials for protection and environmental remediation applications. The porous structure of PAN/PVP aerogels allows for effective containment of spills, while the nanofibers provide a large surface area for the growth of MOFs. Building on our previous findings, we achieved remarkable degradation efficiencies for DMNP, with half‐lives of just 3 minutes and 5 minutes for 2X and 1X mesoporous UiO‐66‐NH_2_ on PAN/PVP aerogels, respectively. Similar trends were observed for nerve agents and blistering agents. The incorporation of mesoporosity using amphoteric surfactants like CAPB enhances mass transfer and significantly improves the degradation efficiency of OPs and nerve agents. Beyond chemical performance, these aerogels exhibit impressive mechanical properties, as demonstrated by DMA and cyclic compression tests. The 1X‐mesoporous UiO‐66‐NH_2_‐loaded aerogels display nearly elastic behavior up to 50% strain, with 14.5% permanent shape loss, while the 2X‐mesoporous UiO‐66‐NH_2_ aerogels maintain elasticity up to 70% strain with only 6% shape loss. This mechanical robustness, combined with their lightweight and porous nature, ensures that these materials can perform reliably under practical operating conditions without compromising their structure. The unique structural characteristics of mesoporous MOFs enable higher surface area and pore volume, promoting more effective interactions with target contaminants. This innovative approach opens new avenues for synthesizing highly functional aerogel‐based materials, paving the way for their use in various practical applications, including protective gear, filtration, and catalysis. Our ongoing research aims ultimately to contribute to the development of effective and safe solutions for addressing protection, decontamination and environmental challenges.

## Conflict of Interest

The authors declare no conflict of interest.

## Author Contributions

M.O.A. and M.Z.A.E contributed equally to this work. M.O.A. and M.Z.A.E.: methodology, investigation, analysis, writing–original draft. V.R.: investigation, analysis. J.J.M.: nerve agents degradation tests, writing–review & editing. G.W.P.: writing–review & editing. S.A.K. and G.N.P.: writing–review & editing, supervision. All authors have approved the final version of the manuscript.

## Supporting information



Supporting Information

Supplemental Video 1

Supplemental Video 2

## Data Availability

The data that support the findings of this study are available from the corresponding author upon reasonable request.
