# Supplementary material for: Mechanically Robust Mesoporous UiO‐66‐NH2/Nanofibrous Aerogel for Organophosphonates Detoxification
Source: Adv Sci (Weinh). 2025 Mar 16;12(18):2416540. doi: 10.1002/advs.202416540 (PMC12079414; doi:10.1002/advs.202416540)
Supplement: Supplementary file 1 — Supporting Information [file ADVS-12-2416540-s003.pdf]

ADVANCED SCIENCE
Open Access

Supporting Information

for *Adv. Sci.*, DOI 10.1002/advs.202416540

Mechanically Robust Mesoporous UiO-66-NH_2/Nanofibrous Aerogel for Organophosphonates Detoxification

Mai O. Abdelmigeed, Muhammed Ziauddin Ahmad Ebrahim, Vahid Rahmanian, John J. Mahle, Gregory W. Peterson, Saad A. Khan and Gregory N. Parsons**

Supporting Information

Mechanically Robust Mesoporous UiO-66-NH_2/Nanofibrous Aerogel for Organophosphonates Detoxification

Mai O. Abdelmigeed, [a] Muhammed Ziauddin Ahmad Ebrahim, [a] Vahid Rahmanian,[a] John J. Mahle, [b] Gregory W. Peterson, [b] Saad Khan, *[a] Gregory N. Parsons*[a]

Materials

All reagents were purchased from commercial sources and used without further purification. Zirconium dichloride oxide hydrate ($ZrOCl_2$. xH_2O, Alfa Aesar, 99.9%), 2-Aminoterephthalic acid (BDC-NH_2, Sigma-Aldrich, 99%), cocamidopropylbetaine surfactant (CAPB, cosmetic grade), acetic acid ($C_2H_4O_2$, ACROS organics, 99.7+%), methanol (Fisher Chemical), N-ethyl morpholine (Sigma-Aldrich, ≥97%), and dimethyl 4-nitrophenyl phosphate (DMNP, Sigma-Aldrich). Polyvinylpyrrolidone (PVP, average M.W. 1,300,000) was obtained from Acros Organics. Polyacrylonitrile (PAN, average M.W 150,000), anhydrous tertiary butanol (t-BuOH, 99+%), dimethylformamide (DMF), dimethylacetamide (DMAC), acetone (99%), hydrochloric acid (HCl, 37%), tetraethoxysilane (TEOS), and isopropanol (> 99.5%) were obtained from Sigma Aldrich. Cellulose diacetate (CDA, acetyl: 39.8% and hydroxyl: 3.5%) was supplied by Eastman Chemicals. Deionized water was used wherever required throughout the experiments. All reagents were used as received without further purification.

Atomic Layer Deposition (ALD) of TiO_2: ALD was used to create thin conformal inorganic TiO_2 coatings on the PAN/PVP nanofibrous aerogels. These samples are referred to as PAN/PVP@TiO_2. TiO_2 was deposited directly on the PAN/PVP nanofibrous aerogels. The fiber coating and analysis procedures follow methods developed previously for ALD modification of polymers and textile media. The ALD TiO_2 was deposited onto PAN/PVP nanofibrous aerogels using a lab-made hot-wall viscous-flow vacuum reactor.

Methodology

Electrospinning of PAN/PVP Nanofibers: The polymer nanofibers used for fabricating NFAs were prepared through a two-step process. First, PAN/PVP nanofibers were synthesized by dissolving 10 wt.% of PAN in DMF, followed by continuous stirring to ensure complete dissolution. Next, an amount of PVP equal to the mass of PAN was added to the solution and stirred thoroughly until fully dissolved. The prepared solution was then transferred into a 10 mL syringe and was mounted onto a syringe pump, with the needle connected to the positive terminal of a high-voltage power supply, while the grounded collector plate served as the counter electrode. The aspect ratio of electrospun nanofibers, a key factor in aerogel structure, was controlled by adjusting polymer concentration, viscosity, applied voltage, flow rate, and collector distance. Higher voltages and lower flow rates produced thinner fibers with increased aspect ratios. Detailed parameter optimization is discussed in our earlier studies.[1] Electrospinning was conducted at 20 kV under ambient room temperature, with a steady flow rate of 0.5 mL/hr, and the distance between the needle tip and the collector set to 15 cm.

Fabrication of NFAs via homogenization followed by freeze-drying: The nanofibrous aerogels were prepared by cutting nonwoven mats into small pieces (~1 × 1 cm^2). About 270 mg of these pieces were immersed in tert-butanol (t-BuOH) as a non-solvent and homogenized at high shear (13,000 rpm)

for 15 minutes, producing a uniform dispersion of finely chopped fibers. Optimizing shear rate during high-speed homogenization and duration ensured uniform aspect ratios, enhancing ifiber entanglement and mechanical stability. The resulting dispersion was transferred to a freeze-drying tray and frozen at -50 °C for 2 hours. After freezing, the system was placed under vacuum at 0.003 mbar, allowing the solvent to sublimate over 48 hours. The collected aerogels were then thermally treated in an oven, first at 180 °C for 2 hours (to stabilize the PAN) and subsequently at 220 °C for an hour (cyclization of the PAN) then used for further experiments.

Sol–Gel Electrospinning of silica/CDA Nanofibers: A cellulose diacetate (CDA) solution (11 wt.%) was prepared by stirring CDA in a DMAC/acetone cosolvent (1:2 by weight) overnight. Separately, a silica precursor mixture was made by combining TEOS, DMF, H2O, and HCl in a 1:10:18.75:0.021 molar ratio, stirred at 70 °C for 1 hour. This silica mixture was then added to the CDA solution in varying ratios and stirred for another hour at the same temperature. For electrospinning, the combined solution was loaded into a 5 mL syringe with a 22-G needle, set to a feed rate of 0.5 mL/h. Electrospinning was conducted at 20 kV, with a 15 cm distance between the needle and the collector, at 23 ± 2 °C and 45 ± 2% humidity. The fibers were collected as a nonwoven membrane on grounded aluminum foil.

Silica/CDA NFA Fabrication: Nanofiber aerogels were produced by freeze-drying nanofiber dispersions in tert-butanol. A 250 mg nanofiber mat was cut into 1x1 cm^2 pieces and dispersed in 20 mL of tert-butanol, then homogenized for 20 minutes at 13,000 rpm. The dispersion was poured into Teflon molds, frozen in a dry ice and 2-propanol mixture, and freeze-dried at <50 Pa and −50 °C for 48 hours to obtain CDA and CDA-silica hybrid nanofiber aerogels (CDA-NFA and hybrid NFA). Finally, the NFA was heated at 180 °C for 2 hours, then at 240 °C for an additional hour in a vacuum oven, resulting in a thermally treated hybrid NFA, referred to as hybrid NFA-therm.

1/2X-Mesoporous UiO-66-NH_2 MOF synthesis: Cocamidopropyl Betaine surfactant (CAPB) (500 mg) was dissolved in deionized water (5 ml) as well as acetic acid (0.9 ml) was added and stirred for 10 minutes to have a homogeneous solution. $ZrOCl_2$. $8H_2O$ (161 mg) and BDC-NH_2 (90.5 mg) were added to the previous mixture and stirred for 10 minutes. The mixture was heated to 60°C for 24 hours under static conditions. The resulting powder was filtered, washed with water 3 times, and methanol 2 times to remove the surfactant, followed by soaking in methanol for four days (replace the methanol soaking solution every 12 hours). The product was dried at 75°C overnight, followed by the activation step at 85°C in a vacuum oven overnight.

1X-Mesoporous UiO-66-NH_2 MOF synthesis: Cocamidopropyl Betaine surfactant (CAPB) (500 mg) was dissolved in deionized water (5 ml) as well as acetic acid (0.9 ml) was added and stirred for 10 minutes to have a homogeneous solution. $ZrOCl_2$. $8H_2O$ (322mg) and BDC-NH_2 (181 mg) were added

to the previous mixture and stirred for 10 minutes. The mixture was heated to 60^oC for 24 hours under static conditions. The resulting powder was filtered, washed with water 3 times, and methanol 2 times to remove the surfactant, followed by soaking in methanol for four days (replace the methanol soaking solution every 12 hours). The product was dried at 75^oC overnight, followed by the activation step at 85^oC in a vacuum oven overnight.

2X-Mesoporous UiO-66-NH_2 MOF synthesis: Cocamidopropyl Betaine surfactant (CAPB) (500 mg) was dissolved in deionized water (5 ml) as well as acetic acid (0.9 ml) was added and stirred for 10 minutes to have a homogeneous solution. $ZrOCl_2$. $8H_2O$ (644mg) and BDC-NH_2 (362 mg) were added to the previous mixture and stirred for 10 minutes. The mixture was heated to 60^oC for 24 hours under static conditions. The resulting powder was filtered, washed with water 3 times, and methanol 2 times to remove the surfactant, followed by soaking in methanol for four days (replace the methanol soaking solution every 12 hours). The product was dried at 75^oC overnight, followed by the activation step at 85^oC in a vacuum oven overnight.

Coating the polypropylene with 20 nm TiO_2 recipe: Deposition pressure was kept at 1.8 Torr, and the temperature was 90 °C. In a general ALD TiO_2 cycle, titanium tetrachloride ($TiCl_4$) was first dosed in the reaction chamber for 1 s, followed by 40 s of N_2 purge between doses. After the $TiCl_4$ dose and N_2 purge, deionized water was dosed with another 40 s of N_2 purge. We chose 300 cycles of ALD TiO_2 as a standard coating thickness onto the fiber mats resulting in 20 nm of coating thickness, as determined by spectroscopic ellipsometer (J. A. Woollam Co., Inc.) on monitor wafers coated simultaneously in the ALD reactor.

For growing 1X-mesoporous UiO-66-NH_2 MOF on aerogel using the solvothermal synthesis technique followed by heat-drying: The same powder recipe mentioned above was followed, but the aerogel was added and sonicated for 10 mins in the solution just before putting this mixture in the oven.

For growing 1X-mesoporous UiO-66-NH_2 MOF on aerogel using the solvothermal synthesis technique followed by freeze-drying: Cocamidopropyl Betaine surfactant (CAPB) (500 mg) was dissolved in deionized water (5 ml) as well as acetic acid (0.9 ml) was added and stirred for 10 minutes to have a homogeneous solution. $ZrOCl_2$. $8H_2O$ (322mg) and BDC-NH_2 (181 mg) were added to the previous mixture and stirred for 10 minutes. Aerogel was added and sonicated for 10 mins in the previous precursors' solution. The mixture was heated to 60^oC for 24 hours under static conditions. The resulting composite was kept submerged and washed with 1 L of deionized water to move the surfactant, followed by soaking in water for one day then freeze-dried in a Labconco FreeZone freeze-drying system at <50 Pa and −50 °C for 48 h.

DMNP hydrolysis: Either 2.5 mg of MOF powder or 14 mg of MOF fabric composite was dispersed in 1 mL of aqueous 0.45 M N-ethylmorpholine (NEM) solution through stirring at 1100 rpm for 15 minutes. Subsequently, 4 μL of DMNP was added, weighed, and dispersed by shaking. The solution was continuously stirred at 1100 rpm and 25 °C. At predefined time intervals, 20 μL samples were extracted and diluted in 10 mL of 0.15 M NEM aqueous solution. The formation of product p-nitrophenoxide was monitored using a Thermo Scientific Evolution 300 UV−vis spectrophotometer at 407 nm. Utilizing DMNP hydrolysis as a characterization technique allowed for direct comparisons between powder and composite materials. The determination of half-life involved linear interpolation between points closest to 50% conversion.

Live nerve agent degradation: Caution! Chemical warfare agents are extremely toxic and should only be handled by trained personnel using appropriate safety protocols. Samples were evaluated for agent reactivity using the following protocol. Materials were activated at ~50 °C for 2 h followed by equilibration at 50% RH overnight. Chemical agent was dosed with ~30 mg activated sorbent samples in a ratio of 1 ul to 10 mg(~10 wt.%). A 24 h incubation step was then performed at room temperature (25 °C). The materials were then extracted using~1.5 mL of acetonitrile for GD tests, isopropyl alcohol for VX tests, and chloroform for HD tests, and vials were chilled in dry ice for GD and HD to quench the reaction and reduce the volatility of any residual agent. The extract was analyzed by gas chromatograph – mass spectrometer and the % removed was calculated based on mass balance from the initial starting volume of the agent.

Characterization

X-ray Diffraction. The crystallographic analysis employed a Rigaku SmartLab X-ray diffractometer with Bragg−Brentano alignment and Cu Kα source. XRD was utilized to assess the crystal structure and the degree of crystallization in MOF powders and composites.

Scanning Electron Microscopy. The Field Emission Scanning Electron Microscope – Hitachi SU8700 and Variable Pressure Scanning Electron Microscope – Hitachi SU3900 were utilized for the analysis. For the SEM analysis, some samples underwent gold/palladium sputtering at 11 mA for 2 minutes. SEM was employed to assess MOF crystal dimensions and morphology, as well as the uniformity of the coating on fibers.

Thermogravimetric Analysis: The oxidative thermal properties of the samples were evaluated using a TA Instrument Discovery SDT 650 in air. The samples were heated at a rate of 10°C/ min, with an isothermal hold at 150°C for one hour to ensure the complete removal of any volatile components.

Transmission Electron Microscopy: Talos F200X G2 is a 200 kV FEG (Field Emission Gun) Analytical Scanning Transmission Electron Microscope (S/TEM).

Fourier-transform infrared spectroscopy (FTIR): Fourier-transform infrared spectroscopy (FTIR) spectra were collected using Thermo scientific, Nicolet iS50 FT-IR.

N_2 Sorption Analysis. Utilizing a Micrometrics 3Flex Physisorption system, nitrogen isotherms were gathered at liquid nitrogen temperatures (−196 °C) and analyzed with Micrometrics 3Flex software. The calculations included determining the Brunauer−Emmett−Teller surface area (BET SA), pore size using density functional theory (DFT), micropore volume at P/P0 = 0.1 (V_{micro}), and total pore volume at P/P0 = 0.98 (V_{tot}). The nitrogen isotherm shape was also employed to confirm the presence of mesopore MOF crystals and MOF-fabric composites. Compressive stress and strain were determined using the following equations, where σ represents stress, ε denotes strain, F is the applied axial force, r is radius of the cylindrical aerogel, h_i is the initial height of the sample, and h_f is the height of the sample at a given time during testing.

Mechanical Properties: Dynamic compression-tension testing was performed using a Discovery Hybrid Rheometer (DHR 3) equipped with a 40 mm parallel plate geometry. The tests involved a series of 10 loading-unloading cycles at each specified strain level, conducted at a constant compression-tension rate of 100 μm/s.

$$\boldsymbol{\sigma = F/\pi r^2}$$

$$\boldsymbol{\varepsilon = \frac{h_i - h_f}{h_i}}$$

Microporosity (%)=

$$\frac{Pore\ Volume\ of\ micropores\ \left(\frac{cm^3}{g}\right)}{Total\ pore\ volume\ \left(\frac{cm^3}{g}\right)} \times 100$$

Mesoporosity (%)=

$$\frac{Pore\ Volume\ of\ mesopores\left(\frac{cm^3}{g}\right)}{Total\ pore\ volume\ \left(\frac{cm^3}{g}\right)} \times 100$$

MOF loading (%) calculated from BET: $\left(\frac{\mathbf{g_{powder}}}{\mathbf{g_{comp}}}\right)$

$$= \frac{Surface\ area\ of\ MOF\text{-}fabric\ composite\ \left(\frac{m^2}{g_{comp}}\right)}{Surface\ area\ of\ MOF\ powder\ \left(\frac{m^2}{g_{powder}}\right)} \times 100$$

Table S.1. Performance Comparison of various UiO-66-NH_2 composite Materials for DMNP hydrolysis.

**Matrix Type**	**Half-life (min) / Conversion (%) of DMNP Hydrolysis**	**MOF loading (wt%)**	Ref
UiO-66-NH2/BPEIH composite	~80% at 15 min reaction time	-	[2]
Graphene/UiO-66-NH2 fabric	3.4 min and >99% conversion in 30 mins	27.7	[3]
MOFs/MIPs/GO	3.11 min/	20	[4]
UiO-66-NH2/cotton fabric	9.2 min	-	[5]
PAN/UiO-66-NH2	5.2 min	37	[6]
PP@TiO2@UiO-66-NH2	4.9	-	[7]
MOF-Cellulose Sponge	9 min	34.5	[8]
UiO-66-NH2/Chitosan/polyamide	92	6.4	[9]
PA-6@PDA@UiO-66-NH2	4	28.4	[10]
PIM/PAN/UiO-66-NH2 Fibrous Mat	70% conversion at 140 min	-	[11]
Dpa@UiO-66-NH2/PAN Fabric	13	56	[11]
MIP/MOF hybrid gel	10 min	25.2	[12]
2X- Mesoporous UiO-66-NH2 on PAN/PVP	3.3 min, Complete conversion:15 mins	73	This Work

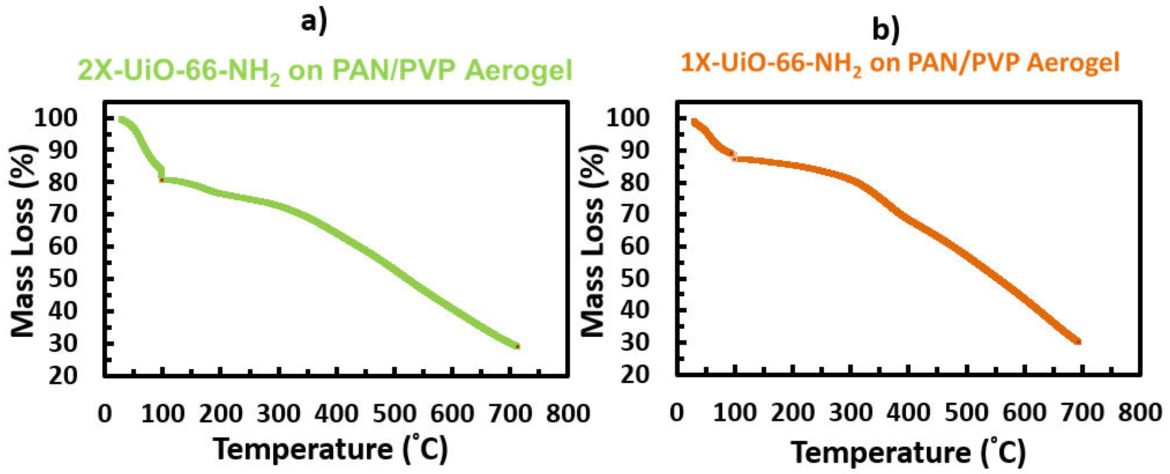

Figure S.1. a) TGA of 2x-mesoporous UiO-66-NH_2 on PAN/PVP nanofibrous aerogel b) TGA of 1x-mesoporous UiO-66-NH_2 on PAN/PVP nanofibrous aerogel

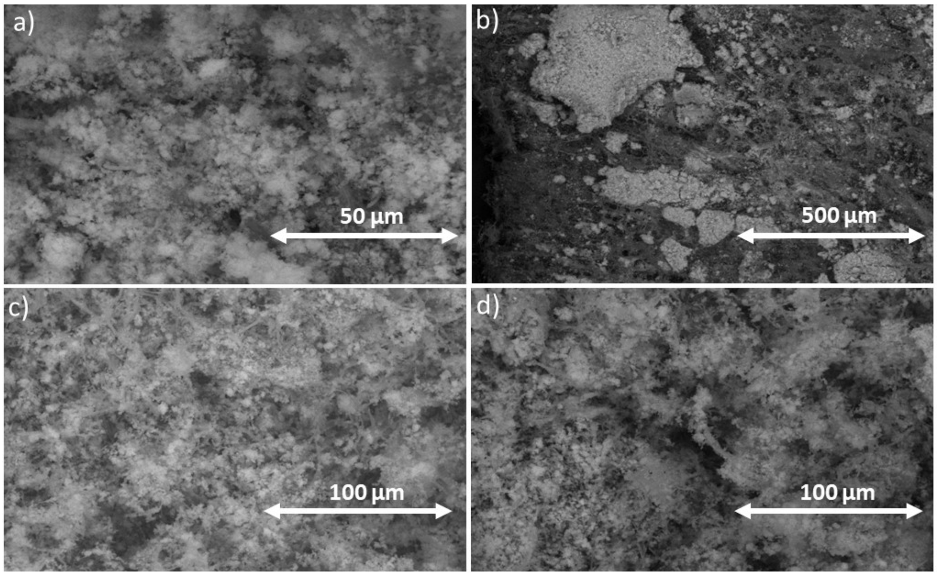

Figure S.2. SEM images of a,b)1X-mesoporous UiO-66-NH_2-PAN/PVP and c,d) 1X-mesoporous UiO-66-NH_2-PAN/PVP@TiO_2 aerogel composites

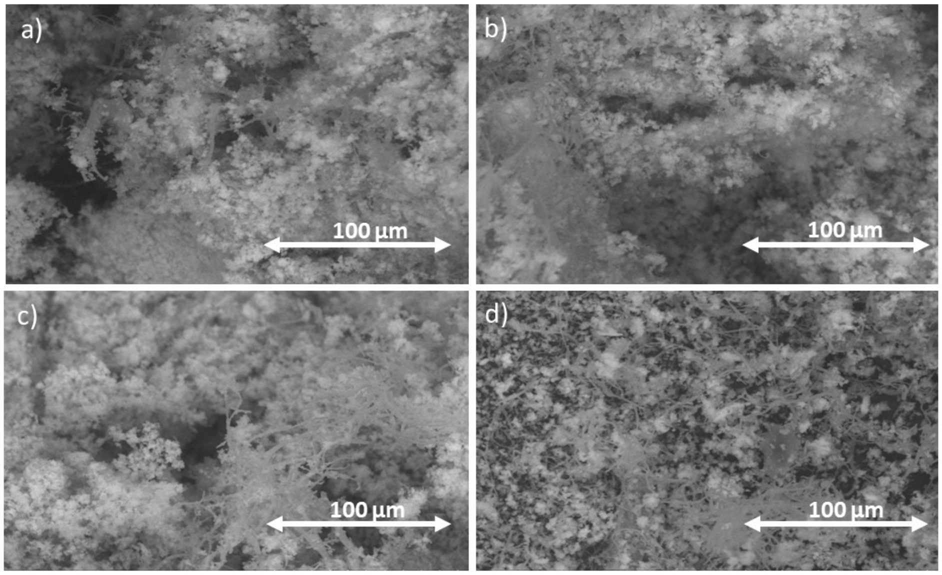

Figure S.3. SEM images of a,b)2X-mesoporous UiO-66-NH_2-PAN/PVP and c,d) 2X-mesoporous UiO-66-NH_2-PAN/PVP@TiO_2 aerogel composites

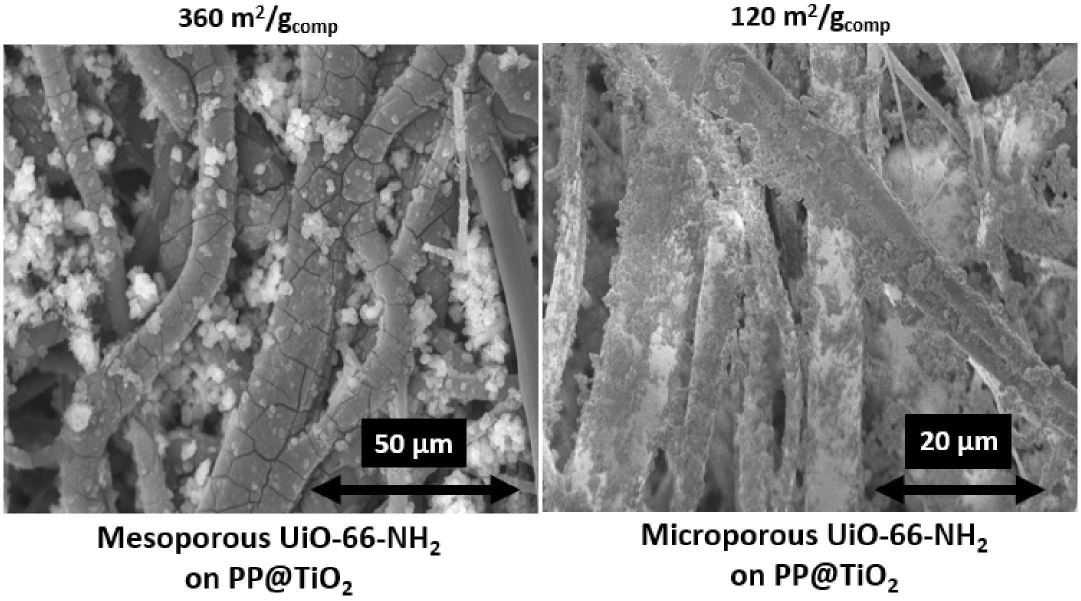

Figure S.4. SEM images of a) mesoporous UiO-66-NH_2 on PP@TiO_2 vs b) microporous UiO-66-NH_2 on PP@TiO_2 composites.[13]

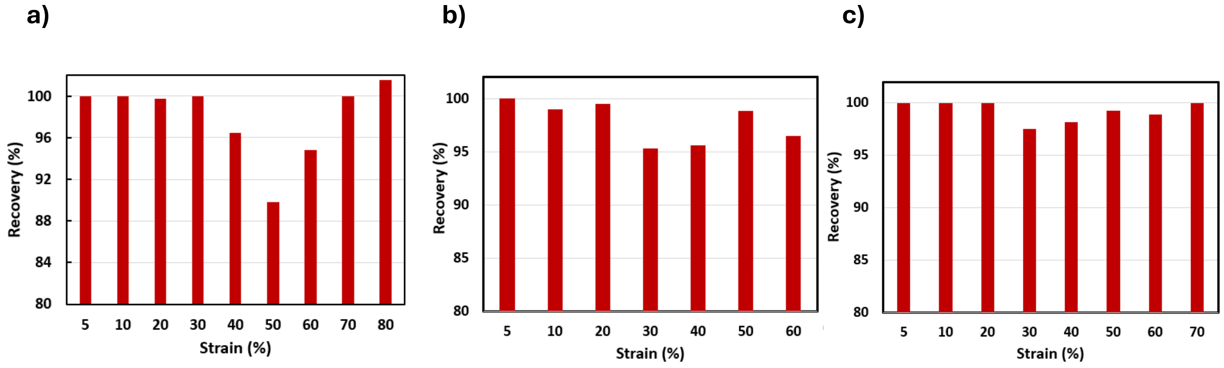

Figure S.5. Dynamic mechanical analysis of: a) PAN/PVP; b) 1X-Mesoporous UiO-66-NH_2 on PAN/PVP; and c) 2X-Mesoporous UiO-66-NH_2 on PAN/PVP.

Spill Containment

A spill containment test was conducted to evaluate the water absorption capacity of PAN/PVP NFAs in comparison to PP. **Figure S.6** shows that PAN/PVP NFAs exhibited a significantly higher absorption capacity, with the ability to absorb 70 grams of water per gram of NFA. In contrast, polypropylene absorbs only 4 grams of water per gram of material. This remarkable difference highlights the superior performance of PAN/PVP NFAs in spill containment applications, attributed to their highly porous structure and increased surface area, which enhances water uptake.

Spill Containment test:

$$\text{Water absorbed}\left(\frac{g_{\text{water}}}{g_{\text{substrate}}}\right) = \frac{mass_{beaker+fibers+water} - mass_{beaker+fibers}}{mass_{fibers}}$$

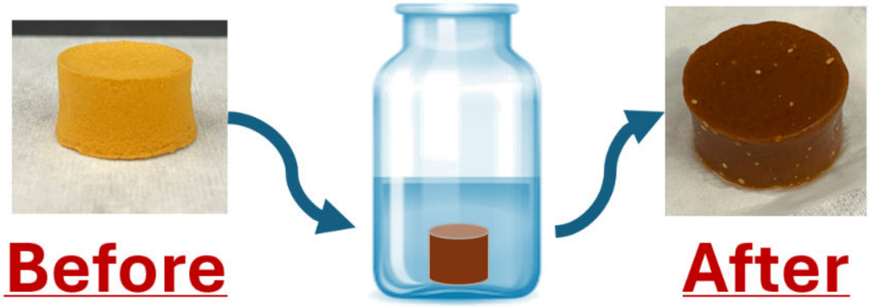

Figure S.6. Spill containment test for PAN/PVP aerogel composites

Solvothermal growth of mesoporous UiO-66-NH_2 on CDA/Silica aerogel

Table S.2 presents the surface area results for mesoporous UiO-66-NH_2/Silica-CDA aerogel composites synthesized via solvothermal techniques followed by ambient drying. These composites showed high surface area, approximately 300 ± 60 m²/g, with 1X-concentrations. However, as shown in **Figure S.7e**, this concentration resulted in brittle composites with diminished mechanical resilience. To address this issue, precursor concentrations were halved, reducing the surface area to around 60 ± 5 m²/g, however, the materials remained brittle remained brittle. The isotherms in **Figures S.7a–d** show a combination of Type I and Type IV characteristics indicating micropores and mesopores within the composites.

Table S.2. BET surface area results of different precursors concentrations of the mesoporous UiO-66-NH_2/Silica-CDA aerogel composites using the solvothermal synthesis technique followed by heat-drying.

#	1X-UiO-66-NH_2 on CDA aerogel	1-X UiO-66-NH_2 on CDA aerogel	1/2X-UiO-66-NH_2 on CDA aerogel	1/2X-UiO-66-NH_2 on CDA aerogel
**BET surface area (m^2/g_{comp})**	**360**	**245**	**55**	**65**

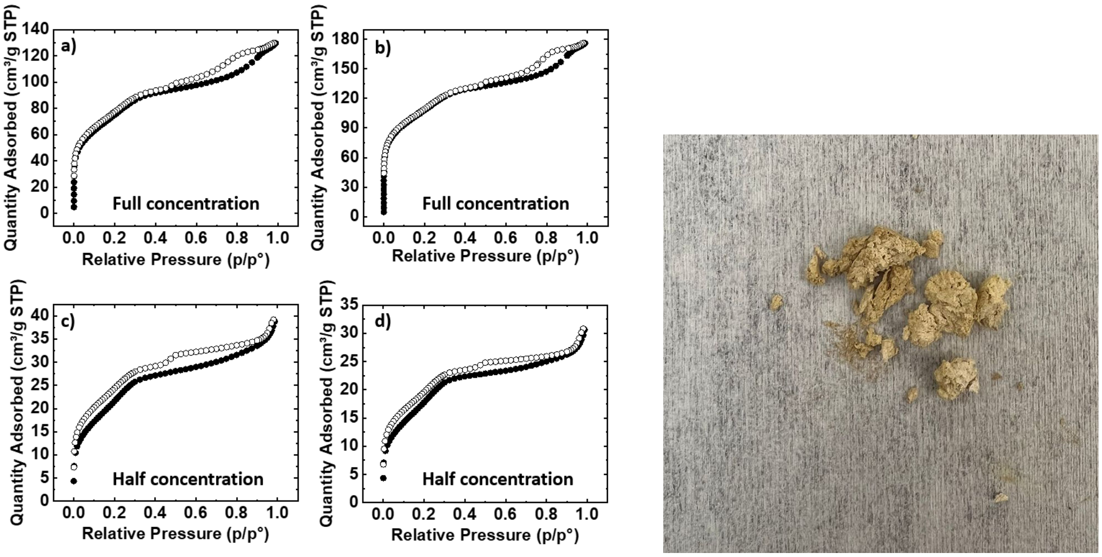

Figure S.7. Isotherms of different precursor's concentrations of the mesoporous UiO-66-NH_2/Silica-CDA aerogel composites using the solvothermal synthesis technique followed by heat-drying.

To address the mechanical degradation of the aerogel composites, an alternative method employed freeze-drying instead of heat-drying. **Table S.3** shows the surface area measurements for mesoporous UiO-66-NH_2/Silica-CDA aerogel composites synthesized through solvothermal methods and subsequently freeze-dried, resulting in a surface area of approximately 170 m²/g. Despite this, the freeze-dried composites exhibited structural softness and collapse, which limited their mechanical stability. **Figure S.8** provides the isotherm for these composites, showing a combination of Type I and Type IV isotherms, indicating both micropores and mesopores.

Table S.3. the BET surface area result of the mesoporous UiO-66-NH_2/Silica-CDA aerogel composite using the solvothermal synthesis technique followed by freeze-drying.

#	1X-UiO-66-NH_2 on CDA aerogel
**BET surface area (m²/g$_{comp}$)**	**167**

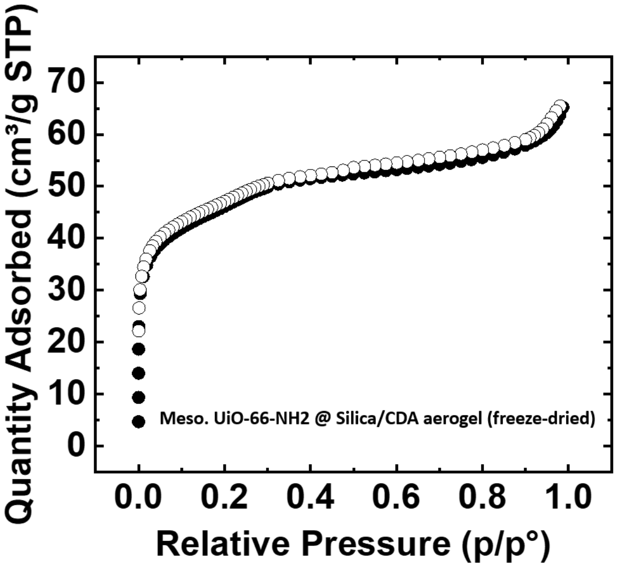

Figure S.8. Isotherm of the mesoporous UiO-66-NH_2/Silica-CDA aerogel composite using the solvothermal synthesis technique followed by freeze-drying.

REFERENCES

[1] M. Z. A. Ebrahim, V. Rahmanian, M. Abdelmigeed, T. Pirzada, S. A. Khan, *Small Methods* **2024**, *8*, 2400596.
[2] K. Ma, M. C. Wasson, X. Wang, X. Zhang, K. B. Idrees, Z. Chen, Y. Wu, S.-J. Lee, R. Cao, Y. Chen, L. Yang, F. A. Son, T. Islamoglu, G. W. Peterson, J. J. Mahle, O. K. Farha, *Chem Catalysis* **2021**, *1*, 721.
[3] L. Song, T. Zhao, D. Yang, X. Wang, X. Hao, Y. Liu, S. Zhang, Z.-Z. Yu, *Journal of Hazardous Materials* **2020**, *393*, 122332.
[4] Y. Niu, P. Jiang, T. Guo, *ACS Appl. Mater. Interfaces* **2024**, *16*, 49305.
[5] Y. Gao, L. Yao, S. Zhang, Q. Yue, W. Yin, *Environmental Pollution* **2023**, *316*, 120622.
[6] R. Xu, T. Wu, X. Jiao, D. Chen, C. Li, *ACS Appl. Mater. Interfaces* **2023**, *15*, 30360.
[7] Highly Breathable Chemically Protective MOF-Fiber Catalysts - Lee - 2022 - Advanced Functional Materials - Wiley Online Library, .
[8] C. Shen, Z. Mao, H. Xu, L. Zhang, Y. Zhong, B. Wang, X. Feng, C. Tao, X. Sui, *Carbohydrate Polymers* **2019**, *213*, 184.
[9] N. Couzon, P. Hardy, M. Ferreira, N. Hammi, J. Dhainaut, F. Pourpoint, S. Royer, T. Loiseau, C. Campagne, C. Volkringer, *Dalton Trans.* **2024**, *53*, 5784.
[10] A. Yao, X. Jiao, D. Chen, C. Li, *ACS Appl. Mater. Interfaces* **2020**, *12*, 18437.
[11] Polymer of intrinsic microporosity (PIM) based fibrous mat: combining particle filtration and rapid catalytic hydrolysis of chemical warfare agent simulants into a highly sorptive, breathable, and mechanically robust fiber matrix - ScienceDirect, .
[12] P. Jiang, Y. Niu, J. Cao, D. Xie, J. Li, T. Guo, *Chemical Engineering Journal* **2024**, *481*, 148377.
[13] M. O. Abdelmigeed, J. J. Mahle, G. W. Peterson, G. N. Parsons, *Small* **2024**, *20*, 2405831.
